# Adverse effects of *Hif1a* mutation and maternal diabetes on the offspring heart

**DOI:** 10.1186/s12933-018-0713-0

**Published:** 2018-05-12

**Authors:** Radka Cerychova, Romana Bohuslavova, Frantisek Papousek, David Sedmera, Pavel Abaffy, Vladimir Benes, Frantisek Kolar, Gabriela Pavlinkova

**Affiliations:** 1 Laboratory of Molecular Pathogenetics, Institute of Biotechnology CAS, BIOCEV, Center of Excellence, Prumyslova 595, 25250 Vestec, Czechia; 2 Faculty of Science, Charles University, Prague, Czechia; 3 Institute of Physiology CAS, Prague, Czechia; 4 Institute of Anatomy, First Faculty of Medicine, Charles University, Prague, Czechia; 5 Laboratory of Gene Expression, Institute of Biotechnology CAS, BIOCEV, Vestec, Czechia; 6 EMBL Genomics Core Facility, Meyerhofstr. 1, 69117 Heidelberg, Germany

**Keywords:** Fetal programming, Maternal diabetes, *Hif1a* haploinsufficiency, Echocardiography, Heart remodelling

## Abstract

**Background:**

Epidemiological studies show that maternal diabetes predisposes offspring to cardiovascular and metabolic disorders. However, the precise mechanisms for the underlying penetrance and disease predisposition remain poorly understood. We examined whether hypoxia-inducible factor 1 alpha, in combination with exposure to a diabetic intrauterine environment, influences the function and molecular structure of the adult offspring heart.

**Methods and results:**

In a mouse model, we demonstrated that haploinsufficient (*Hif1a*^+*/*−^) offspring from a diabetic pregnancy developed left ventricle dysfunction at 12 weeks of age, as manifested by decreased fractional shortening and structural remodeling of the myocardium. Transcriptional profiling by RNA-seq revealed significant transcriptome changes in the left ventricle of diabetes-exposed *Hif1a*^+*/*−^ offspring associated with development, metabolism, apoptosis, and blood vessel physiology. In contrast, both *wild type* and *Hif1a*^+*/*−^ offspring from diabetic pregnancies showed changes in immune system processes and inflammatory responses. Immunohistochemical analyses demonstrated that the combination of haploinsufficiency of *Hif1a* and exposure to maternal diabetes resulted in impaired macrophage infiltration, increased levels of advanced glycation end products, and changes in vascular homeostasis in the adult offspring heart.

**Conclusions:**

Together our findings provide evidence that a global reduction in *Hif1a* gene dosage increases predisposition of the offspring exposed to maternal diabetes to cardiac dysfunction, and also underscore *Hif1a* as a critical factor in the fetal programming of adult cardiovascular disease.

**Electronic supplementary material:**

The online version of this article (10.1186/s12933-018-0713-0) contains supplementary material, which is available to authorized users.

## Background

Parallel to the increasing global incidence of diabetes, the prevalence of diabetes in women of childbearing age is steadily rising. Diabetic pregnancy has been associated with a higher risk of adverse outcomes for the mother as well as the offspring compared to non-diabetic pregnancy [1–4]. Congenital heart defects are the most common malformations observed in offspring of diabetic pregnancies, with an eightfold increase compared to non-diabetic pregnancies [5]. Similar risks for cardiac malformation have been reported for pre-gestational type 1 or type 2 *diabetes mellitus* [5]. Since type 1 and type 2 diabetes have different etiologies, these results indicate that the adverse effects on heart development are induced by pathological processes shared by both types of diabetes, such as hyperglycemia, hypoxia, oxidative stress, and abnormal maternal/fetal fuel metabolism.

In addition to the direct teratogenicity of maternal diabetes, the intrauterine and early postnatal environment can influence the cardiovascular and metabolic health of offspring later in life. This phenomenon is termed fetal or developmental programming [6]. The offspring of a diabetic pregnancy show differences in metabolic, cardiovascular and inflammatory variables compared to the offspring of non-diabetic mothers [7–9]. However, the precise mechanisms for the underlying penetrance and disease predisposition remain poorly understood.

Hypoxia plays an important role in all diabetic complications [10–12], including the complications associated with diabetic pregnancy [2, 13–15]. The main regulator of responses to a hypoxic environment is hypoxia-inducible factor 1 (HIF-1). HIF-1 consists of two subunits, HIF-1α, which is an O_2_-labile subunit, and HIF-1β, which is constitutively expressed [16]. HIF-1α is also important for normal embryonic development since mice with a homozygous deletion of the *Hif1a* gene die due to cardiac malformations and vascular defects [17]. *Hif1a* heterozygous mutants (*Hif1a*^+*/*−^) normally survive past embryonic development; however, *Hif1a*^+*/*−^ mice demonstrate impaired responses when challenged with hypoxic conditions after birth [18–20]. Cardiac myocyte-specific *Hif1a* deletion causes reductions in contractility, vascularization, and also alters the expression of multiple genes in the heart during normoxia [21]. HIF-1α is destabilized by hyperglycemia leading to the loss of cellular adaptation to low oxygen in diabetes [10, 22]. Previously, we showed that decreased levels of *Hif1a* in combination with a diabetic environment were associated with increased susceptibility to diabetic embryopathy [23]. We found a decreased number of embryos per litter and increased incidence of heart malformations, particularly atrioventricular septal defects and reduced myocardial mass in diabetes-exposed *Hif1a*^+*/*−^ mice compared to *wild type* (*Wt*) littermates. To extend our previous study on the effects of global heterozygous deletion of *Hif1a* and maternal diabetes exposure on heart development in embryos [23], we analysed the heart of the adult offspring in the same experimental paradigm. We examined the relationship between a partial deficiency of HIF-1α and an intrauterine exposure to maternal diabetes in the fetal programming of the heart.

## Research design and methods

### Animals

All animal experiments were approved by the Animal Care and Use Committee of the Institute of Molecular Genetics and performed in compliance with the Guide for the Care and Use of Laboratory Animals (National Institutes of Health publication, 8th edition, updated 2011). Diabetes was induced in female inbred FVB mouse strain (*Wt*, strain code 207, Charles River), aged 7–9 weeks, by 2 intraperitoneal injections of 100 mg/kg body weight of streptozotocin (STZ; Sigma) within a 1-week interval, as described [23]. Blood glucose levels were measured in animals by glucometer (COUNTOUR TS, Bayer); blood glucose levels maintained above 13.9 mmol/L are classified as diabetic. The dams were set up for mating no earlier than 7 days after the last injection. Average blood glucose level of STZ-induced female *Wt* before mating was 18.54 ± 1.08 mmol/L. This experimental design ensured that the development of the embryos proceeded in the intrauterine environment of maternal diabetes. Only diabetic *Wt* females were mated to *Hif1a*^+*/*−^ males with the *Hif1a*^*tm1jhu*^ null allele [17] on an FVB background to generate *Hif1a*^+*/*−^ and *Wt* (*Hif1a*^+*/*+^) offspring (n = 11 litters of non-diabetic pregnancy, n = 10 litters of diabetic pregnancy). *Hif1a*^+*/*−^ mice show a partial loss of HIF-1α protein expression levels [18, 20]. Mice were kept under standard experimental conditions with constant temperature (23–24 °C) and fed on soy-free feed (LASvendi, Germany). The females were housed individually during the gestation period and the litter size was recorded. All dams were pregnant for the first time. Offspring of *Wt* female× *Hif1a*^+*/*–^ male mating were genotyped by PCR [23].

### Echocardiography

The echocardiographic evaluation of the geometrical and functional parameters of the left ventricle (LV) was performed using the GE Vivid 7 Dimension (GE Vingmed Ultrasound, Horten, Norway) with a 12 MHz linear matrix probe M12L. The 12-week-old animals were anesthetized by the inhalation of 2% isoflurane (Aerrane, Baxter) and their rectal temperature was maintained between 36.5 and 37.5 **°**C by a heated table throughout the measurements. The 1-week old offspring were measured without anesthesia. The following diastolic and systolic dimensions of the LV were measured: the posterior and anterior wall thickness, and cavity diameter (LVD_D_ and LVD_S_). From these dimensions, the functional parameter, fractional shortening (FS) was derived by the following formula:

$${\text{FS }}\left[ \% \right] = 100 \times \left( {{\text{LVD}}_{\text{D}} - {\text{LVD}}_{\text{S}} } \right)/{\text{LVD}}_{\text{D}} .$$

### RNA-sequencing

Total RNA was extracted from the LV of the hearts of 12-week-old mice. The quality of the isolated RNA was analyzed using Bioanalyzer 2100 (Agilent) and a functional test was performed by reverse transcription (RT) and quantitative real-time PCR (qPCR) using oligodT primers for RT and PCR amplification of a 996-bp product of the *Hprt1* gene. 3′-seq libraries from 3 LVs per group (n = 3 samples from mice from multiple litters/group) were prepared and next-generation sequencing was performed on an Illumina NextSeq sequencer (NextSeq500) using a mode enabling the determination of uni-directional 75 bases and indices, following manufacturer’s instructions at the Genomics Core Facility (EMBL Heidelberg, Germany). The number of reads (minimum, 32 million; maximum, 73 million) was filtered out by TrimmomaticPE version 0.36 [24]. Ribosomal RNA and mitochondrial reads were filtered out by Sortmerna (version 2.1b [25]) using default parameters. RNAseq reads were mapped to the mouse genome using STAR (version 2.5.2b [26]) version GRCm38 primary assembly and annotation version M8. Count table was generated by python script htseq-count (version 0.6.1p1, [27]) with parameter “–m union”. The raw RNAseq data were deposited at GEO (http://www.ncbi.nlm.nih.gov/geo/) #GSE109633. DESeq2 (v. 1.15.51, [28]) default parameters were used to normalize data and compare the different groups. We selected differentially expressed genes based on an adjusted P value < 0.1. Functional classification was done using DAVID Functional Annotation tool (https://david.ncifcrf.gov/). For the Venn diagram, a 30% change threshold was applied in each group compared to unexposed *Wt*. Mammalian phenotype ontology enrichment analysis of MGI (http://www.informatics.jax.org) was used. Enrichment analysis was performed using g:GOST Gene Group Functional Profiling; g:Profiler (http://biit.cs.ut.ee/gprofiler/). Manual literature search and Harmonizome database [29] were used to identify direct HIF-1 targets and predicted HIF-1 target genes, respectively.

### Reverse transcription-quantitative real-time polymerase chain reaction

Total RNA was isolated from the LV of the hearts of offspring at 12 weeks of age (n = 8 samples/group). Following reverse transcription (RT), quantitative real-time PCR (qPCR) was performed with the initial AmpliTaq activation at 95 °C for 10 min, followed by 40 cycles at 95 °C for 15 s and 60 °C for 30 s, as described [19]. The *Hprt1* gene was selected as the best reference gene for our analyses from a panel of 12 control genes (TATAA Biocenter AB, Sweden). The relative expression of a target gene was calculated, based on qPCR efficiencies and the quantification cycle (Cq) difference (Δ) of an experimental sample versus control. Primers were designed using Primer Blast tool (https://www.ncbi.nlm.nih.gov/tools/primer-blast/). Primers were selected according to the following parameters: length between 18 and 24 bases, melting temperature (Tm) between 58° and 60 °C, G+C content between 40 and 60% (optimal 50%) and efficiency above 80%. Primer sequences are presented in Additional file 1: Table S1.

### Western blot assays

The LV from the diabetic and non-diabetic hearts were lysed with protease and phosphatase inhibitors to prevent protein degradation and stored at − 80 °C until analysis. 50 µg of total protein lysates per lane were resolved using 10% SDS-PAGE and transferred to a nitrocellulose membrane. The membrane was blocked with 5% dry milk and incubated overnight with anti-CX43 antibody at 1:6000 (C6219, Sigma), anti-pCX43 at 1:1000 (3511, Cell Signaling), and anti-TNFR2 at 1:1000 (sc-7862, Santa Cruz Biotechnology). After incubation with a horseradish peroxidase-conjugated secondary IgG (Sigma), the blots were developed using the SuperSignal™ West Femto Maximum Sensitivity Substrate (#34095; Thermo Scientific, MI, USA). Chemiluminescent signals were captured using an Biorad Chemidoc MP Imager and analyzed by ImageJ software (http://imagej.nih.gov/ij/download.html). Ponceau S staining was used as the loading control.

### Morphological and immunohistochemical analyses

The scanning electron microscope (SEM) images of 12-week-old mouse hearts were produced as described [30]. Paraffin Sections (8 μm) were stained with Periodic acid–Schiff [PAS; staining of advanced glycation end products (AGEs); 395B, Sigma], Picrosirius Red (collagen staining, 24901-250, Polysciences), TUNEL (#1684795, Roche), anti-PECAM-1 1:50 (Ab28364, Abcam), and anti-VEGFA 1:50 (sc-7269, Santa Cruz Biotechnology). Vibratome Sections (100 μm) were stained with anti-F4/80 1:500 (MCA497R, Biorad). The nuclei were counterstained with Hoechst 33342. The sections were assessed under a Nikon Eclipse 50i and Nikon Eclipse E400 microscopes, Leica MZFLIII stereomicroscope, and Zeiss LSM 880 confocal microscope, with NIS-elements or ZEN lite programs. The number of F4/80^+^ cells were specifically counted in the LV area in the field of view (2–5 Z-stacks/5 areas of the LV/1 vibratome section/4 individuals/group). TUNEL^+^ cells were counted in three consecutive transversal paraffin sections of the heart/4 individuals/group, as described [23]. Picrosirius Red^+^, PAS^+^ and PECAM-1^+^ areas were quantified as a percentage of the LV area in the field of view using Image J software. VEGFA expression was determined specifically in the wall of blood vessels of the LV as a percentage of vessel area using Image J software (three consecutive transversal sections of the heart/4 individuals/group).

### Statistics

We used two-way ANOVA to compare differences among experimental groups with genotype and experimental condition (diabetes or no diabetes exposure) as the two categories. When a significant interaction was detected, the differences between subgroups were further analysed by post hoc Tukey’s multiple comparison and *t* tests; significance assigned at the P < 0.05 level (GraphPad Prism 7).

## Results

### Exposure to maternal diabetes and *Hif1a* deficiency alter heart function in offspring

**Fig. 1:**
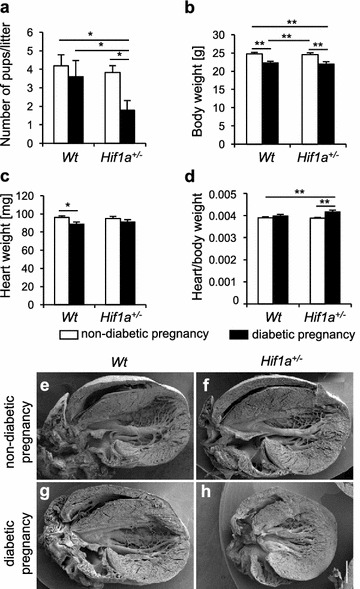
Adverse effects of maternal diabetes exposure and *Hif1a* mutation on the survival, and heart weight of the offspring. Average number of surviving *Hif1a*^+*/*−^ pups per litter from diabetic pregnancy at P7 compared to non-diabetic pregnancy (**a**). The body weight (**b**), heart weight (**c**), and heart/body ratio (**d**) in all groups of 12-week-old *Wt* and *Hif1a*^+*/*−^ mice from non-diabetic and diabetic pregnancies. The values are mean ± SEM (**a** n = 11 litters of non-diabetic pregnancy, n = 10 litters of diabetic pregnancy; **b**–**d** n = 20 *Wt* from non-diabetic pregnancy, n = 15 *Wt* from diabetic pregnancy, n = 15 for *Hif1a*^+*/*−^ from non-diabetic pregnancy, n = 10 *Hif1a*^+*/*−^ from diabetic pregnancy). Statistical significance assessed by two-way ANOVA: diabetes effect (number of pups/litter, P = 0.0391; body weight, P < 0.0001; heart weight, P = 0.0124; heart/body weight, P = 0.0003); and interaction between genotype and diabetes (heart/body weight, P = 0.0362). *Significant differences by post hoc Tukey’s multiple-comparison test, *P < 0.05, **P < 0.01. **e**–**h** Representative scanning electron micrographs (SEM) of the posterior half of the 12-week-old mouse heart

In our previous study, deletion of *Hif1a*^+*/*−^ in combination with maternal diabetes was shown to lead to an increased incidence of congenital cardiac defects [23]. Consistent with the previous study, we found the number of liveborn *Hif1a*^+*/*−^ offspring from a diabetic pregnancy (n = 10 litters) significantly decreased compared to the number of *Wt* littermates from a diabetic pregnancy or the number of offspring from a non-diabetic pregnancy (n = 11 litters; Fig. 1a). To minimize the potential influence of maternal genotype, *Hif1a*^+*/*−^ offspring were generated by mating *Wt* females with *Hif1a*^+*/*−^ males. Body weight was decreased in both *Wt* and *Hif1a*^+*/*−^ offspring exposed to maternal diabetes, whereas the heart weight was lower in *Wt* mice at 12 weeks of age (Fig. 1b, c). The heart to body weight ratio, an index of cardiac enlargement, was significantly increased as a result of the combined effect of *Hif1a* mutation and fetal exposure to maternal diabetes (Fig. 1d). The gross morphology of the hearts revealed no differences in the structure of chambers or valves (Fig. 1e–h). Interestingly, the heart of diabetes-exposed *Hif1a*^+*/*−^ offspring had a globular shape with less elongated and more globular ventricles compared to the hearts in others groups of adult offspring. The globular shape of the diabetes-exposed *Hif1a*^+*/*−^ heart was indicated by the ratio of the left–right axis (width) and the apical-basal axis (length) of the heart (mean ratio = 1.03) compared to the normal shaped hearts of unexposed *Wt* and *Hif1a*^+*/*−^, and diabetes-exposed *Wt* (mean ratio = 1.38, 1.39, and 1.38, respectively).

**Fig. 2:**
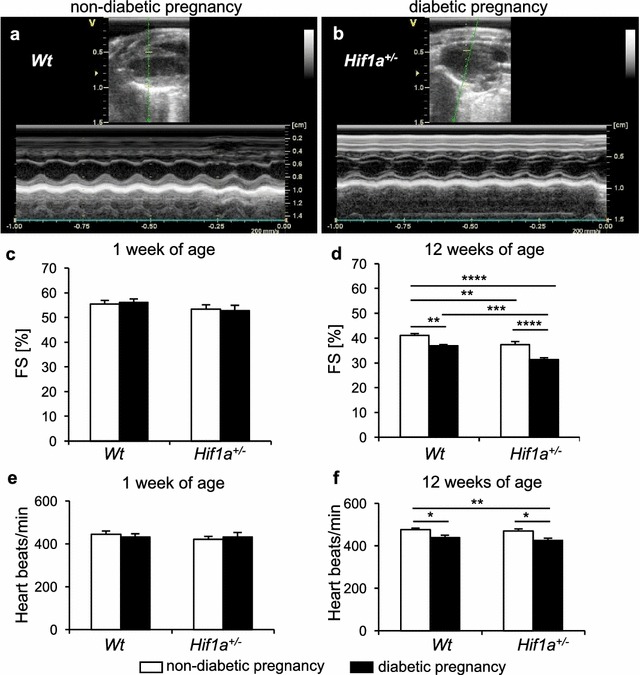
The combination of *Hif1a* haploinsufficiency and maternal diabetes exposure alters echocardiographic parameters of the offspring. Representative M-mode recordings of LV structures in long axes view in **a** *Wt* males from non-diabetic pregnancy and **b** *Hif1a*^+*/*−^ males from diabetic pregnancy. **c**, **d** The echocardiographic evaluation of LV systolic function, fractional shortening (FS), in the 1- and 12-week-old male offspring. **e**, **f** The heart rate in *Wt* and *Hif1a*^+*/*−^ males from both diabetic and non-diabetic pregnancies at 1 and 12 weeks of age. The values are mean ± SEM (1-week-old: n = 20 *Wt* from non-diabetic pregnancy; n = 14 *Wt* from diabetic pregnancy; n = 17 *Hif1a*^+*/*−^ from non-diabetic pregnancy; n = 9 *Hif1a*^+*/*−^ from diabetic pregnancy; 12-week-old: n = 20 *Wt* from non-diabetic pregnancy; n = 15 *Wt* from diabetic pregnancy and *Hif1a*^+*/*−^ from non-diabetic pregnancy; n = 10 *Hif1a*^+*/*−^ from diabetic pregnancy). Statistical significance assessed by two-way ANOVA: genotype effect (FS% P < 0.001); and diabetes effect (FS% P < 0.0001; heart rate, P < 0.0001). *Significant differences by post hoc Tukey’s multiple-comparison test, *P < 0.05, **P < 0.01, ***P < 0.001, ****P < 0.0001

Echocardiographic analyses of the LV function in 1- and 12-week-old offspring disclosed a significant decrease in fractional shortening (FS) in diabetes-exposed *Hif1a*^+*/*−^ compared to diabetes-exposed *Wt* (P < 0.001), and unexposed *Wt* or *Hif1a*^+*/*−^ controls (P < 0.0001) at 12 weeks of age (Fig. 2a–d, Additional file 2: Table S2). These changes were associated with *Hif1a*^+*/*−^ genotype and with diabetes exposure, predominantly as a result of a larger systolic diameter of the LV of diabetes-exposed *Hif1a*^+*/*−^ mice (Additional file 2: Table S2). Both males and females were similarly affected in LV echocardiographic parameters (Additional file 2: Table S2), therefore, we used only males for our subsequent analyses. Both diabetes-exposed *Wt* and *Hif1a*^+*/*−^ males had a lower heart rate at 12 weeks of age (Fig. 2e, f), which may indicate changes in cardiovascular autonomic regulation [31].

### Differential global expression profiles in the LV of the diabetes-exposed *Hif1a*^+*/*−^ and *Wt* offspring

**Fig. 3:**
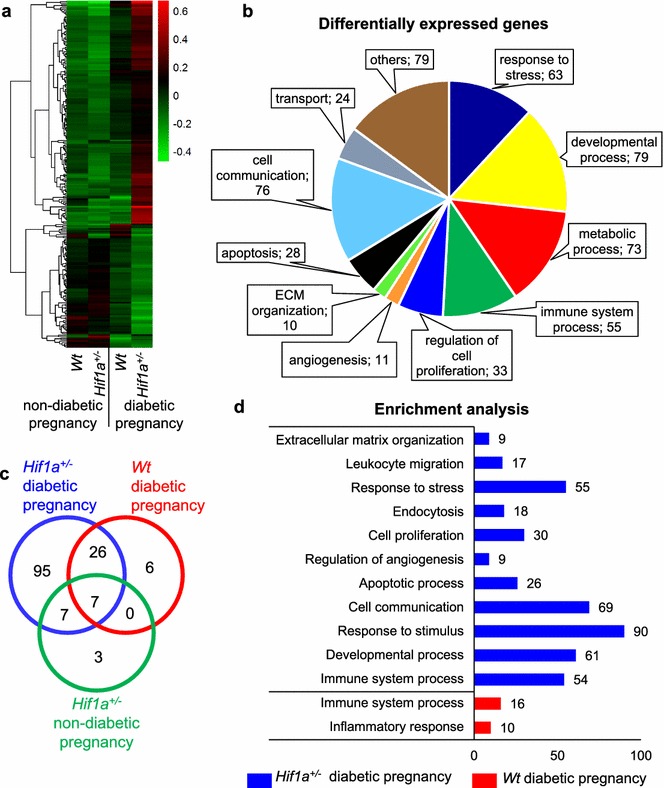
Maternal diabetes and *Hif1a* mutation affect the transcriptome of the LV of *Hif1a*^+*/*−^ offspring. The global gene expression profile of LVs isolated from the hearts of 12-week-old *Wt* and *Hif1a*^+*/*−^ mice from diabetic or non-diabetic pregnancy (n = 3 LVs from mice of different litters per group) was determined by RNA sequencing. **a** Hierarchical clustering of differentially expressed genes in all groups are presented as a heat map and dendrogram. **b** Classification of differentially expressed genes affected by *Hif1a*^+*/*−^ mutation and exposure to maternal diabetes by functional annotation tool DAVID. Values represent number of genes per each category. **c** Venn diagram shows the number and proportion of differentially (up- and down-regulated) expressed genes with ≥ 30% change in *Hif1a*^+*/*−^ and *Wt* from diabetic pregnancy and *Hif1a*^+*/*−^ from non-diabetic pregnancy *vs. Wt* from non-diabetic pregnancy. **d** Enrichment analysis using g:Profiler; values represent number of genes per each biological process category

To map out the mechanisms underlying impaired cardiac function mediated by the exposure to maternal diabetes, we analyzed the expression profiles of the LV from the hearts of offspring using RNA deep sequencing (RNAseq). The expression profiles visualized in a heat map indicate major changes in the *Hif1a*^+*/*−^ offspring from diabetic pregnancies (Fig. 3a) with categories of genes relevant to metabolic processes, cell communication, apoptosis, immune system, and developmental processes (Fig. 3b, Additional file 3: Table S3). We focused on differentially expressed genes showing ≥ 30% change in any of the groups compared to *Wt* from non-diabetic pregnancy, representing 144 differentially expressed genes; the majority of changes (95 genes) were identified only in the diabetes-exposed *Hif1a*^+*/*−^ hearts (Fig. 3c, Additional file 4: Table S4, Additional file 5: Table S5). Of 135 differentially expressed genes in diabetes-exposed *Hif1a*^+*/*−^ hearts, 53% of these genes were direct or predicted HIF-1 target genes (Additional file 6: Table S6, Additional file 7: Table S7). Enrichment analysis revealed changes in immune system processes and inflammatory response in both *Wt* and *Hif1a*^+*/*−^ offspring from diabetic pregnancies (Fig. 3d, Additional file 8: Table S8). However, the *Hif1a*^+*/*−^ offspring from a diabetic pregnancy showed additional changes in multiple categories of biological processes associated with development, response to stress, apoptosis, cell proliferation and communication, and angiogenesis. Mammalian phenotype ontology enrichment analysis revealed significant changes in genes predominantly associated with homeostasis and metabolism phenotype (63 genes, P = 6.93 × 10^−4^), immune system phenotype (60 genes, P = 2.74 × 10^−7^), and abnormal innate immunity (25 genes, P = 3.93 × 10^−10^, Additional file 9: Table S9). Additionally, 17 genes were associated with abnormal blood vessel physiology (P = 5.85 × 10^−6^) and vascular smooth muscle physiology (9 genes, P = 2.573 × 10^−3^). We validated RNAseq data by RT-qPCR for a selected set of genes (Additional file 10: Figure S1).

### Cardiac structural remodelling in the diabetes-exposed offspring

**Fig. 4:**
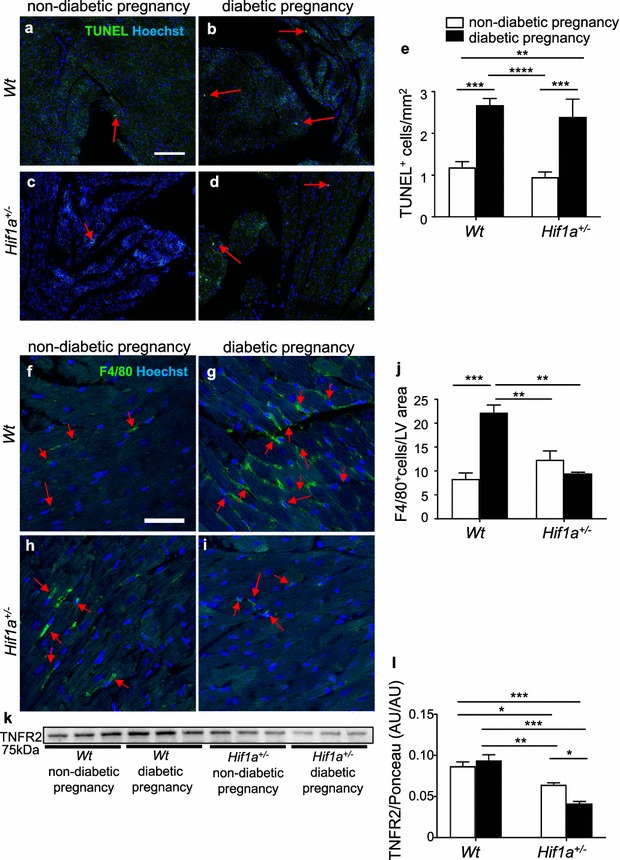
Maternal diabetes exposure increases apoptosis and *Hif1a*^+*/*−^ mutation alters macrophage infiltration in the LV myocardium of the 12-week old offspring. **a**–**d** Representative images of TUNEL staining (green) with Hoechst stained cell nuclei (blue) in adult myocardium (arrows indicate TUNEL^+^ cells). Scale bar = 100 µm. **e** Quantification of TUNEL^+^ apoptotic cells per mm^2^ of the LV myocardium. The values are mean ± SEM (n = 4 individuals/3 sections/group). **f**–**i** Representative confocal images of immunohistochemical staining of macrophage marker F4/80 (green) with Hoechst stained cell nuclei (blue) in adult myocardium (arrows indicate F4/80^+^ cells). Scale bar = 50 µm. **j** Quantification of F4/80^+^ cells per area in the field of view in LV myocardium. The values are mean ± SEM (n = 1 section/2–5 z-stacks/4 individuals/group). **k**, **l** Representative Western blot and quantification of TNFR2 expression in the LV myocardium. The values are mean ± SEM (n = 3). Two-way ANOVA indicating interaction between genotype and diabetes (F4/80, P = 0.0002; TUNEL, P = 0.016; TNFR2, P = 0.0133) genotype effect (F4/80, P = 0.0159); diabetes effect (F4/80, P = 0.0042) followed by post hoc Tukey’s multiple-comparison test, *P < 0.05, **P < 0.01, ***P < 0.001, ****P < 0.0001. *TNFR2* tumor necrosis factor receptor type II, *AU* arbitrary units

Impaired LV function may be associated with myocardial remodelling, including increased cell death. We assessed the number of apoptotic cells in the LV (Fig. 4a–e), right ventricle (RV), and septum (Additional file 11: Figure S2). We found increased apoptosis in the RV and LV myocardium, and in the septum of both *Hif1a*^+*/*−^ and *Wt* diabetes-exposed offspring.

Since macrophages play a key role in myocardial injury repair and remodelling [32], we analysed the recruitment of macrophages. In contrast to increased apoptosis in both diabetes-exposed *Hif1a*^+*/*−^ and *Wt,* the number of F4/80^+^ infiltrating macrophages was significantly increased only in the LV of the diabetes-exposed *Wt* heart (Fig. 4f–j). Correspondingly to decreased macrophage infiltration, we found a significantly reduced level of TNFR2 in the LV of diabetes-exposed *Hif1a*^+*/*−^ mice (Fig. 4k, l). TNFR2 is a cardio-protective mediator of TNFα signalling and its deletion results in reduced macrophage infiltration [33, 34].

**Fig. 5:**
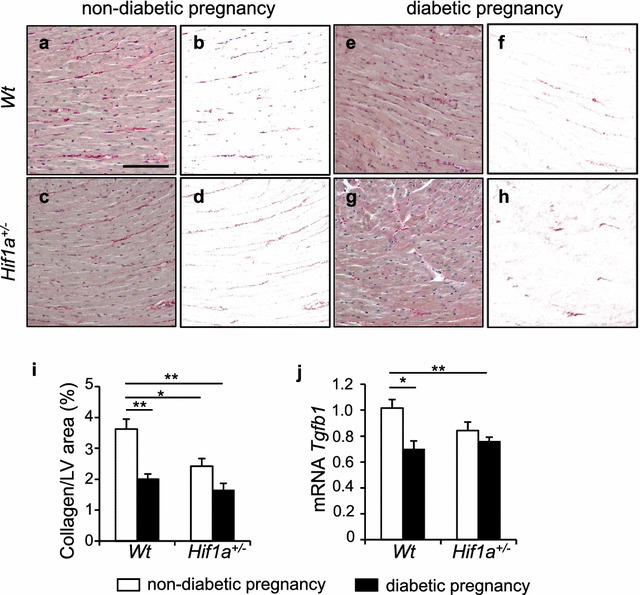
Remodeling of extracellular matrix in the offspring heart is induced by the exposure to maternal diabetes. Representative images of picrosirius red staining of collagen in the LV myocardium and delineated collagen^+^ area in the myocardium using Adobe Photoshop (**a**–**h**). Scale bar = 100 µm. **i** Relative quantification of staining determined as a percentage of collagen^+^ areas per tissue area of the LV myocardium in the field of view by ImageJ. The values are means ± SEM (n = 4). **j** Relative mRNA expression levels of *Tgfb1* in the LV myocardium by RT-qPCR, the values are mean ± SEM (n = 8/group). Data are normalized to *Hprt1* mRNA of control gene. Statistical significance assessed by two-way ANOVA: genotype effect (tissue collagen, P = 0.0089); diabetes effect (tissue collagen, P = 0.0007; *Tgfb1*, P = 0.0079) followed by *t* tests, *P < 0.05, **P < 0.01. *Tgfb1* Transforming growth factor beta 1

The composition of the myocardial extracellular matrix (ECM) influences cardiac structure and hemodynamic functions. Our RNAseq analysis indicated transcriptional changes in genes encoding ECM structural proteins and ECM regulators. The major component of the ECM is collagen, predominantly collagen type I [35]. Surprisingly, we found decreased collagen deposition in both diabetes-exposed *Hif1a*^+*/*−^ and *Wt* and in unexposed *Hif1a*^+*/*−^ LVs when compared to *Wt* (Fig. 5a–i). Increased collagen deposition and pro-fibrogenic processes have been linked to the activation of transforming growth factor ß (TGF-ß) signalling in the heart [36]. The expression pattern of *Tgfb1* mRNA, which is the most prevalent isoform of TGF-ß, was similar to the deposition pattern of collagen (Fig. 5j).

**Fig. 6:**
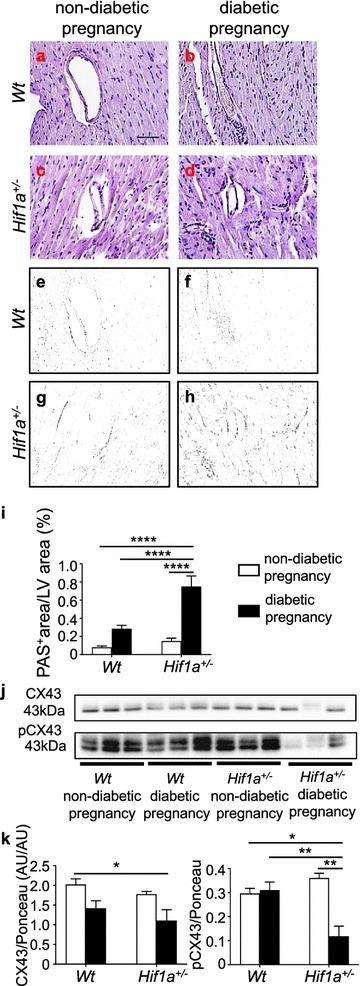
The amount of AGEs is increased whereas the activity of gap junction CX43 is reduced in the heart of diabetes-exposed *Hif1a*^+*/*−^ offspring. Representative images of PAS staining of advanced glycation end products (AGE; **a**–**d**). Scale bar = 50 µm. **e–h** Delineated PAS^+^ area in the myocardium by Adobe Photoshop. **i** Quantification of PAS staining determined as a percentage of positive area in the field of view by ImageJ. The values are mean ± SEM (n = 4). Representative Western blots and quantification of both CX43 and its phosphorylated isoform pCX43 in the LV myocardium of the offspring (**j**, **k**). The values are mean ± SEM (n = 3). Statistical significance assessed by two-way ANOVA: diabetes effect (CX43, P = 0.0088) and interaction between genotype and diabetes (AGEs, P = 0.0013; pCX43, P = 0.0030) followed by post hoc Tukey’s multiple-comparison test, *P < 0.05, **P < 0.01, ****P < 0.0001. *CX43* connexin 43, *PAS* periodic acid-shiff, *AU* arbitrary units

To further assess the effect of tissue-exposure to hyperglycemia, we analyzed the accumulation of advanced glycation end products (AGEs) in the heart. The diabetic environment increases the production of AGEs, which comprise adverse non-enzymatic tissue modifications of proteins, lipids, and nucleic acids [37]. Interestingly, we detected a significant increase of AGEs in the LV of the *Hif1a*^+*/*−^ offspring of a diabetic pregnancy (Fig. 6a–i). AGEs have been implicated in the development of cardiac dysfunction by altering properties of the extracellular matrix. Therefore, the increased production of AGEs in the diabetes-exposed *Hif1a*^+*/*−^ heart may represent an increased risk for vascular and myocardial damage in association with impaired contractility, inflammation, and endothelial dysfunction.

The remodelling of gap junctions and changes in the expression of connexin 43 (CX43) have been associated with a diseased myocardium [38]. CX43 is a key mediator of cardiomyocyte protection during hypoxia [39] and CX43 expression in hypoxia is regulated by HIF-1α [40]. Additionally, the expression and proportion of the phosphorylated and dephosphorylated forms of CX43 are altered in diabetic conditions [41]. In our study, the relative abundance of CX43 protein was reduced in the LV of *Hif1a*^+*/*−^ offspring from a diabetic pregnancy compared to the offspring from a non-diabetic pregnancy (Fig. 6j, k). CX43 phosphorylated at serine 368 (pCX43) was significantly reduced in the diabetes-exposed *Hif1a*^+*/*−^ offspring compared to other groups. Phosphorylation of CX43 is important for the functionality of this protein in gap junctions [42] and reduced levels of phosphorylated CX43 have been shown to correlate with worsened heart function in the heart failure model [43].Thus, decreased levels of phosphorylated CX43 in the heart may negatively influence the myocardial function of *Hif1a*^+*/*−^ mice from diabetic pregnancies.

### Changes in genes encoding molecules important for cardiac metabolism

**Fig. 7:**
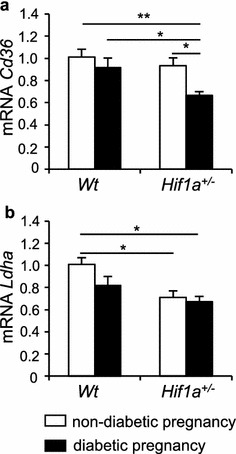
*Hif1a* mutation and maternal diabetes exposure alter the expression of metabolic genes in the LV myocardium. Relative mRNA expression levels of *Cd36* (**a**) and *Ldha* (**b**) in the LV myocardium of 12-week-old offspring by RT-qPCR, the values are mean ± SEM (n = 8). Data are normalized to *Hprt1* mRNA of control gene. Statistical significance assessed by two-way ANOVA: genotype effect (*Cd36* mRNA, P = 0.0118; *Ldha*, P = 0.0034); and diabetes effect (*Cd36* mRNA, P = 0.0061) with post hoc Tukey’s multiple-comparison test, *P < 0.05, **P < 0.01

Alterations in myocardial substrate selection and utilization in energy metabolism play a role in the development of cardiac pathologies [44]. Our RNAseq analyses showed large changes in the expression of genes associated with metabolic processes. In keeping with this, we investigated two HIF-1 target genes, *Cd36* [45] and *Ldha,* gene encoding lactate dehydrogenase A [46]. CD36 is a multifunctional receptor mediating the uptake of lipoproteins and lipoprotein-derived fatty acid (FA) by cardiomyocytes [47]. LDHA catalyzes the terminal step of anaerobic glycolysis, which is the conversion of pyruvate to lactate. We found decreased *Cd36* mRNA in the diabetes-exposed *Hif1a*^+*/*−^ offspring compared to other groups (Fig. 7a). *Ldha* expression was significantly decreased in both diabetes-exposed and non-exposed *Hif1a*^+*/*−^ hearts (Fig. 7b). Altogether, our data suggest metabolic cardiac remodelling associated with *Hif1a* deficiency and maternal diabetes exposure.

### Combination of diabetes pregnancy and *Hif1a* deficiency alters vascular homeostasis in the myocardium

**Fig. 8:**
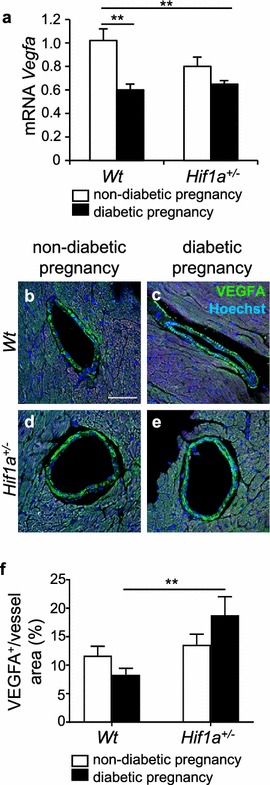
Changes in the expression of *Vegfa* in the heart of the diabetes-exposed offspring. Relative mRNA levels of *Vegfa* in the LV myocardium of the heart of 12-week-old offspring by RT-qPCR (**a**), the values are mean ± SEM (n = 8). Data are normalized to *Hprt1* mRNA of control gene. **b**–**e** Representative confocal images of immunohistochemical staining of VEGFA (green) in the wall of coronary vessels in the LV myocardium of the 12-week-old offspring, Hoechst stained cell nuclei (blue). Scale bar = 50 µm. **f** A relative quantification of VEGFA in the blood vessels is determined as a percentage of VEGFA^+^ area per blood vessel area. The values are mean ± SEM (n = 4). Statistical significance assessed by two-way ANOVA: diabetes effect (*Vegfa* mRNA, P = 0.0003) and interaction between genotype and diabetes (VEGFA in blood vessels, P = 0.0382) followed by post hoc Tukey’s multiple-comparison test, *P < 0.05, **P < 0.01

Because maternal diabetes has been associated with an increased risk for the development of cardiovascular abnormalities in offspring [7], we specifically analyzed the expression of vascular endothelial growth factor (*Vegfa),* a key HIF-1 target gene. The expression of *Vegfa* mRNA was decreased in the LV of both *Hif1a*^+*/*−^ and *Wt* offspring of diabetic pregnancies (Fig. 8a). Using PECAM-1, an endothelial marker, we did not detect any significant changes, indicating comparable microvasculature in the LV of all offspring groups (Additional file 12: Figure S3). Using immunohistochemistry, we evaluated VEGFA expression in the large blood vessels of the LV myocardium. In contrast to the levels of *Vegfa* mRNA in the LV, VEGFA expression in the blood vessel wall was significantly higher in diabetes-exposed *Hif1a*^+*/*−^ than in *Wt* hearts (Fig. 8b–f). This surprising finding indicates that *Vegfa* expression is regulated differently in the myocardium and blood vessel wall. In agreement with our results, a similar expression profile of *Vegfa* was reported in the myocardium and macrovascular tissues of diabetic patients [48]. Thus, our data provide evidence of differential regulation of *Vegfa* in cardiac tissue and coronary macrovasculature of the diabetes-exposed *Hif1a*^+*/*−^.

## Discussion

In this study, we uncovered a molecular mechanism for the underlying penetrance and disease predisposition in the offspring associated with exposure to maternal diabetes. The combination of *Hif1a* insufficiency and exposure to diabetes in utero leads to the accelerated development of cardiac LV dysfunction. RNAseq analysis showed changes in the global expression profile of the LV of *Hif1a*^+*/*−^ heart, indicating transcriptional reprogramming as a consequence of exposure to maternal diabetes. This reprogramming was associated with major changes in HIF-1 regulated pathways, as 53% of identified differentially expressed genes were direct and predicted HIF-1 targets. Thus, the combination of maternal diabetes and *Hif1a* haploinsufficiency results in significant metabolic, structural, and functional changes in the LV myocardium of the offspring.

Both human and animal studies have shown that exposure to diabetes in utero increases cardiovascular risk factors in the offspring [5, 7, 8, 49]. In agreement with these reports, our study showed that exposure to maternal diabetes during the fetal and perinatal period compromised cardiovascular function of the adult offspring, as indicated by smaller fractional shortening. For the first time to our knowledge, the current study demonstrated that *Hif1a* haploinsufficiency significantly worsens the cardiac function of the diabetes-exposed offspring. Additionally, we identified an unknown gene-environment interaction between a genetic deficiency in *Hif1a* and maternal diabetes that affects the size and shape of the heart of the *Hif1a*^+*/*−^ offspring. The globular cardiac shape has been observed in children with fetal growth restriction and associated with systolic dysfunction as a result of the intrauterine chronic hypoxia-induced changes in cardiac development [50].

Maternal diabetes negatively affects embryonic development and growth, and compromises placental function [5, 13, 51]. Although here we focused on the heart of the adult offspring, due to the global nature of the *Hif1a* deletion, we cannot exclude the contribution of placental dysfunction to the programmed outcome phenotype. HIF signalling, in particular, plays an important role in the development and functions of the placenta, and hypoxia-induced placental pathologies have been associated with fetal programming [15, 52–54].

Our genome-scale transcriptome analyses clearly identified a set of pathophysiological processes affected by the maternal diabetes exposure and *Hif1a*^+*/*−^ genotype in the LV of offspring. Specifically, we found changes predominantly in metabolic processes, alterations in the innate and adaptive immune responses, apoptosis, and changes in genes associated with developmental processes. In contrast, the *Wt* offspring from diabetic pregnancies show only significant enrichment of genes involved in immune system processes and inflammatory responses. Clinical studies imply that women with gestational diabetes are at a higher risk of cardiovascular diseases in association with inflammatory dysregulation [3, 4, 55, 56]. Correspondingly, we found an increased number of infiltrating F4/80^+^ macrophages in the LV of *Wt*, but not in *Hif1a*^+*/*−^ offspring of diabetic pregnancies (Fig. 4). Altered macrophage migration in the *Hif1a*^+*/*−^ myocardium is consistent with a recent finding that HIF-1α has an essential role in macrophage inflammatory responses in other physiological settings [32]. The observed increase in TUNEL staining suggests that reduced macrophage recruitment to *Hif1a*^+*/*−^ hearts may lead to decreased phagocytosis of apoptotic cells, which may negatively affect the maintenance of tissue integrity and lead to impaired heart function [32].

Beside apoptosis, interstitial fibrosis is another important process of cardiac ventricular remodeling, contributing to both systolic and diastolic contractile dysfunction. Collagen secretion is stimulated by TGF-ß1 within the heart [57]. Although increased cardiac fibrosis is a hallmark of structural remodelling in response to a diabetic environment [58], we paradoxically found decreased collagen deposition along with decreased expression of *Tgfb1* in both *Hif1a*^+*/*−^ and *Wt* diabetes-exposed LV at 12 weeks of age (Fig. 5). Diminished collagen deposition was also detected in the hearts of *Hif1a*^+*/*−^ offspring from a non-diabetic pregnancy (Fig. 5). HIF-1 regulates multiple steps in collagen biogenesis and HIF-1α knockdown results in reduced collagen deposition [59]. Consistent with this finding, *Hif1a* haploinsufficiency may affect collagen levels in the heart of *Hif1a*^+*/*−^ mice. Similarly, the diabetic environment destabilizes HIF-1α and alters HIF-1 regulation [10, 22] that may affect collagen metabolism in the heart of offspring, resulting in the reduction of collagen fibrils. However, investigating the link between in utero exposure to maternal diabetes and cardiovascular outcomes in offspring is complicated by the multitude of factors that may be present at different time points. Moreover, fibrotic responses are affected by age and duration of disease, when the decreased levels of collagen may represent an intermediate stage of cardiac remodelling associated with degradation of the extracellular collagen matrix [60]. Thus, disturbance in the synthetic and degradative aspects of collagen metabolism results in profound structural and functional abnormalities of the heart. A loss of collagen fibrils is associated with LV remodelling during volume overload and with ECM remodelling in dilated cardiomyopathy [61]. Decreased collagen expression has been reported in children with failing single ventricle congenital heart disease [62]. Although, based on our current data, we cannot determine whether reduced collagen content represents adverse ECM remodelling that predisposes to cardiac dysfunction, our results clearly indicate that the exposure to maternal diabetes and *Hif1a* haploinsufficiency significantly affect ECM structure and composition compared with a normal myocardium.

Another mechanism implicated in the pathophysiology of the diabetic environment is enhanced production of AGEs. In the heart, protein glycation reactions alter the physiological properties of extracellular matrix proteins and cause intracellular changes in vascular and myocardial tissue [37]. As such, AGEs represent a cardiovascular risk factor in the development of macro- and microvascular complications in diabetic patients [63]. The increased levels of these pro-oxidant diabetogenic products correlate with increased oxidative stress, inflammation, and apoptosis in diabetic animals as well as in their progeny [64]. We found a significant accumulation of AGEs in the LV of *Hif1a*^+*/*−^ offspring from a diabetic pregnancy (Fig. 6). These results provide new insights into the role of HIF-1α haploinsufficiency in susceptibility to enhanced accumulation of AGEs due to maternal diabetes exposure. Given that (i) HIF-1 regulates glucose metabolism and glycolysis to minimize oxidative stress [16], (ii) diabetic *Hif1a*^+*/*−^ mice compared to diabetic *Wt* have increased serum glucose and AGEs [65], and impaired glucose homeostasis [66], it is tempting to speculate that deficiency in HIF-1 regulation during embryonic development results in increased AGEs in the cardiac tissue of *Hif1a*^+*/*−^ offspring due to systemic changes in glucose metabolism and oxidative stress in the maternal diabetes environment. To clarify this question raised by our model, detailed analyses of the levels of tissue AGE modifications during embryonic and early postnatal development are now needed.

Besides structural remodelling, we identified changes in the expression of genes associated with metabolic processes that may contribute to impaired cardiac performance and increase the risk of developing heart disease in the offspring from diabetic pregnancy. Alterations in myocardial energy substrate are predominantly represented by changes in the ratio of fatty acid oxidation and glucose oxidation and have been associated with the development of cardiac pathologies [44]. During perinatal cardiac development, the heart undergoes a switch in energy substrate preference from glucose in the fetal period to FAs. In the diabetic environment, the fetal heart is exposed to abnormal levels of substrates that may remodel cardiac metabolism of the offspring. We found significantly decreased expression of gene encoding the fatty acid translocase, *Cd36*, in the heart of *Hif1a*^+*/*−^ offspring from a diabetic pregnancy compared to other groups (Fig. 7). Decreased myocardial levels of CD36 have been found detrimental, even in the absence of elevated circulating FAs, and to contribute to cardiac dysfunction [67]. The decreased *Cd36* expression observed in the hearts of *Hif1a*^+*/*−^ offspring of diabetic pregnancies is consistent with a previous report that *Cd36* expression is regulated by HIF-1 [45]. Expression of another direct HIF-1 target, the glycolytic gene *Ldha* [68], was significantly reduced in the LV by *Hif1a* haploinsufficiency (Fig. 7). Since optimal bioenergetics are an important prerequisite for contractile efficiency, any abnormalities resulting in decreased energy production, energy transfer and energy utilization may compromise cardiac function.

To further investigate changes in angiogenesis, indicated by our RNAseq, we analysed the expression of VEGFA in the heart of the offspring. We detected a decrease in cardiac *Vegfa* mRNA in the LV of both *Hif1a*^+*/*−^ and *Wt* offspring from diabetic pregnancies (Fig. 8). Indeed, several reports have demonstrated that the expression of *Vegfa* mRNA and protein are decreased in the myocardium of diabetic, insulin-resistant animals, and in diabetic patients, and have been associated with vascular abnormalities in the diabetic heart and with diabetic cardiomyopathy [69, 70]. Cardiomyocyte-specific *Vegfa* deletion demonstrates that cardiac myocytes are a major source of VEGFA in the heart and that the development and maintenance of coronary macrovasculature are compensated by non-cardiomyocyte *Vegfa* expression [71]. This phenotype also implies a different signalling mechanism for vasculogenesis/angiogenesis in the myocardium and in the coronary vasculature. In line with these data, we detected a different *Vegfa* expression pattern in the cardiac tissue and in the wall of large coronary vessels. VEGFA levels in the coronary vessels were increased by the combination of *Hif1a* haploinsufficiency and diabetes exposure. A similar expression profile with reduced *Vegfa* levels in the cardiac tissue and increased *Vegfa* expression in macrovascular tissues was reported in diabetic patients [48]. Thus, these paradoxical changes in the expression of *Vegfa* suggest that local regulatory factors differ between the myocardium and blood vessels. It is conceivable that pathophysiological conditions, such as diabetes or hypoxia, alter the regulation of *Vegfa* expression in the coronary macrovasculature and myocardium.

## Conclusions

In a mouse model of maternal diabetes exposure, HIF-1α heterozygous loss-of-function was associated with impaired cardiac function and structural reprogramming of the heart of offspring, including decreased macrophage migration, increased accumulation of AGEs, and altered *Vegfa* expression. Since the HIF-1 system is compromised in the diabetic environment, a failure to adequately express and activate HIF-1α regulation provide a molecular mechanism that may contribute to the cardiac dysfunction seen in the offspring. Furthermore, the frequencies of single-nucleotide polymorphisms of the human *HIF1A* gene, which are associated with reduced HIF-1 activity, were significantly increased in patients with ischemic heart disease presentation [72]. Taken together, our results are compelling evidence of the role of HIF-1α-controlled pathways in increasing the risk of cardiovascular diseases in the offspring of diabetic mothers.

## Additional files

**Additional file 1: Table S1.** Primer sequences for RT-qPCR.

**Additional file 2: Table S2.** Basal left ventricular echocardiographic parameters, heart rate, and body weight of the offspring.

**Additional file 3: Table S3.** Bioinformatics classification of differentially expressed genes.

**Additional file 4: Table S4.** List of genes in Venn diagram in Fig. 3.

**Additional file 5: Table S5.** List of differentially expressed genes with fold change ≥ 30%.

**Additional file 6: Table S6.** The list of HIF-1 signaling target genes.

**Additional file 7: Table S7.** The list of the references for genes linked to HIF-1 signalling by manual literature search.

**Additional file 8: Table S8.** Bioinformatics enrichment analysis using gProfiler: g:GOST Gene Group Functional Profiling tool.

**Additional file 9: Table S9.** Bioinformatics mammalian phenotype ontology enrichment analysis using MouseMine Analysis Tools.

**Additional file 10: Figure S1.** RNAseq data validation by RT-qPCR. The values are mean ± SEM (n = 3/group for RNA-Seq, n = 8/group for RT-qPCR).

**Additional file 11: Figure S2.** Quantification of TUNEL^+^ apoptotic cells per mm^2^ of the RV myocardium (**a**) and septum (**b**). The values are mean ± SEM (n = 4 individuals/3 sections/group). Two-way ANOVA indicating a significant effect of diabetes (RV: P < 0.0001; septum: P < 0.0001) followed by post hoc Tukey’s multiple-comparison test, **P < 0.01, ***P < 0.001, **** P < 0.0001.

**Additional file 12: Figure S3.** PECAM-1 expression in the LV. Representative images of staining of PECAM-1 (red) with Hoechst stained cell nuclei (blue) in the LV of 12 week-old offspring (**a-d**). Scale bar = 50 μm. **e-h**: Delineated PECAM+ area in the myocardium using Adobe Photoshop. Quantification of PECAM-1 staining determined as a percentage of positive area in the field of view by ImageJ (**i**). The values are mean ± SEM (n = 4). Statistical significance assessed by two-way ANOVA: genotype effect P = 0.0302, followed by post hoc Tukey’s multiple-comparison test with no significant result.

## Abbreviations

AGEs: advanced glycation end products
CD36: cluster of differentiation 36 (fatty acid translocase)
COL1: collagen 1
CX43: connexin 43
FA: fatty acid
FS: fractional shortening
HIF-1α: hypoxia-inducible factor 1 alpha
LDHA: lactate dehydrogenase A
LV: left ventricle
PAS: periodic acid-shiff
RNAseq: RNA deep sequencing
RT-qPCR: reverse transcription-quantitative real-time polymerase chain reaction
RV: right ventricle
STZ: streptozotocin
TGFß1: transforming growth factor, beta 1
TNFα: tumor necrosis factor
TNFR2: tumor necrosis factor receptor type II
TUNEL: terminal deoxynucleotidyl transferase dUTP nick end labeling
VEGFA: vascular endothelial growth factor A
Wt: wild type
