# Supplementary material for: Adverse effects of Hif1a mutation and maternal diabetes on the offspring heart
Source: Cardiovasc Diabetol. 2018 May 12;17:68. doi: 10.1186/s12933-018-0713-0 (PMC5948854; doi:10.1186/s12933-018-0713-0)
Supplement: Supplementary file 1 — Additional file 1: Table S1. Primer sequences for RT-qPCR. [file 12933_2018_713_MOESM1_ESM.pdf]

Table S1. Primer sequences for RT-qPCR

**Gene name**	**sequence 5'→3'**
Hprt1 **F**	GCTTGCTGGTGAAAAGGACCTCTCGAAG
Hprt1 **R**	CCTGAAGTACTCATTATAGTCAAGGGCAT
Axl **F**	CATCCTCAAGGTCGCTGTGA
Axl **R**	CATGACGTTGGGGTGGTCAA
Ccl9 **F**	AGCCTTTTCATACTGCCCTCT
Ccl9 **R**	CAATTTCAAGCCCTTGCTGTG
Cd248 **F**	GCAGCCTTACAGGGGTACAG
Cd248 **R**	TGTGGTCTCAGCCATGTGTC
CD36 **F**	GCTCGTTTCAACTCTCACACAC
CD36 **R**	TACGTGGCCCGGTTCTACTA
Cfp **F**	CCATGTAGTCCCAACGCCAC
Cfp **R**	TCTCACCCTGACCTTCAACC
Fbn1 **F**	GCAGAAACACGATTGGCTCC
Fbn1 **R**	GTTCCCGTTTCCAGTTGCAC
Herc3 **F**	TTGAAGGAACTGTCACCAACTG
Herc3 **R**	TGTCTTCACCGGGGTAATCT
Icam **F**	GAGACGCAGAGGACCTTAACA
Icam **R**	ACAGTTACTTGGCTCCCTTCC
Kdm3a **F**	GGGAATGTCAACAAGGAGAATAA
Kdm3a **R**	TTGTTCAGAGGGGGCAAG
LDHa **F**	GCACTGACGCAGACAAGG
LDHa **R**	TGATCACCTCGTAGGCACTG
Lrp1 **F**	ACTGGACTAACTGGCACACG
Lrp1 **R**	CGATACCCCTCGGCATCTTC
Msr1 **F**	GGGAAGTGGATAAATCAGTGCT
Msr1 **R**	CCTCTTGTTCATGAGGGCAGA
Mylk3 **F**	ACTTTGGTAGGGTCCACATTCC
Mylk3 **R**	CTAGAGGCGTCAGCTTGCAC
Pi16 **F**	TGGCACGAGGAGCATGAGTA
Pi16 **R**	GGGAGCCACAGCCAATTCTC
Tgfb1 **F**	GCTGAACCAAGGAGACGGAA
Tgfb1 **R**	ATGTCATGGATGGTGCCCAG
Tgfbr2 **F**	CGATTACATCATCTTTTCGGAAG
Tgfbr2 **R**	TGGACACGGTAGCAGTAGAAGA
Timp1 **F**	GGCATCTGGCATCCTCTTGT
Timp1 **R**	TGGTCTCGTTGATTTCTGGGG
Vegfa **F**	ACTGGACCCTGGCTTTACTG
Vegfa **R**	TGGGACTTCTGCTCTCCTTC
