# Supplementary material for: Adverse effects of Hif1a mutation and maternal diabetes on the offspring heart
Source: Cardiovasc Diabetol. 2018 May 12;17:68. doi: 10.1186/s12933-018-0713-0 (PMC5948854; doi:10.1186/s12933-018-0713-0)
Supplement: Supplementary file 2 — Additional file 2: Table S2. Basal left ventricular echocardiographic parameters, heart rate, and body weight of the offspring. [file 12933_2018_713_MOESM2_ESM.pdf]

Table S2. Basal left ventricular echocardiographic parameters, heart rate, and body weight of the offspring.

1-week-old male offspring

group/(n)	AWTd	LVDd	PWTd	AWTs	LVDs	PWTs	FS %	HR	BW
Wt **non-diabetic pregnancy/(n=20)**	0.37	1.92	0.39	0.62	0.86	0.67	55. 5	444	3.7
**SEM**	0.01	0.03	0.01	0.02	0.03	0.01	1.4	16	0.09
Wt **diabetic pregnancy/(n=14)**	0.4	1.69	0.4	0.56	0.74	0.65	56.3	431	2.8
**SEM**	0.01	0.06	0.01	0.02	0.04	0.03	1.24	16	0.19
Hif1a$^{+/-}$ **non-diabetic pregnancy/(n=17)**	0.4	1.99	0.41	0.62	0.93	0.68	53.5	421	3.7
**SEM**	0.01	0.03	0.01	0.02	0.04	0.02	1.68	14	0.1
Hif1a$^{+/-}$ **diabetic pregnancy/(n=9)**	0.37	1.74	0.38	0.55	0.83	0.59	52.9	431	3.0
**SEM**	0.01	0.1	0.01	0.02	0.07	0.03	2.05	22	0.253

12-week-old male offspring

group/(n)	AWTd	LVDd	PWTd	AWTs	LVDs	PWTs	FS %	HR	BW
Wt **non-diabetic pregnancy/(n=20)**	0.76	3.92	0.7	1.17	2.31	1.22	41.2†‡§	477†§	25.9†§
**SEM**	0.02	0.06	0.02	0.02	0.05	0.02	0.6	6	1.5
Wt **diabetic pregnancy/(n=15)**	0.69	3.73	0.66	1.02	2.35	1.06	37.0*§	440*	23.6*‡
**SEM**	0.01	0.04	0.01	0.02	0.04	0.02	0.4	10	0.6
Hif1a$^{+/-}$ **non-diabetic pregnancy/(n=15)**	0.75	3.93	0.70	1.10	2.46	1.13	37.5*§	469§	27.6†§
**SEM**	0.02	0.09	0.02	0.03	0.09	0.03	1.1	10	0.8
Hif1a$^{+/-}$ **diabetic pregnancy/(n=10)**	0.62	3.85	0.61	0.91	2.64	0.93	31.4*†‡	425*‡	24.0*‡
**SEM**	0.01	0.04	0.01	0.02	0.03	0.03	0.6	10	0.7

1-week-old female offspring

group/(n)	AWTd	LVDd	PWTd	AWTs	LVDs	PWTs	FS %	HR	BW
Wt **non-diabetic pregnancy/(n=19)**	0.36	1.87	0.38	0.60	0.83	0.64	55.6	439	3.6
**SEM**	0.01	0.03	0.01	0.02	0.04	0.02	1.47	14	0.10
Wt **diabetic pregnancy/(n=18)**	0.39	1.75	0.39	0.61	0.79	0.64	54.9	427	3.1
**SEM**	0.01	0.05	0.01	0.01	0.03	0.02	1.14	12	0.15
Hif1a$^{+/-}$ **non-diabetic pregnancy/(n=22)**	0.38	1.90	0.40	0.62	0.83	0.67	56.1	429	3.4
**SEM**	0.01	0.03	0.01	0.02	0.02	0.02	1.07	13	0.11
Hif1a$^{+/-}$ **diabetic pregnancy/(n=7)**	0.39	1.71	0.40	0.57	0.77	0.61	54.9	416	3.0
**SEM**	0.01	0.06	0.01	0.01	0.04	0.02	1.44	31	0.21

12-week-old female offspring

group/(n)	AWTd	LVDd	PWTd	AWTs	LVDs	PWTs	FS %	HR	BW
Wt **non-diabetic pregnancy/(n=22)**	0.67	3.72	0.65	1.08	2.29	1.09	38.1†‡§	419	22.8†§
**SEM**	0.01	0.03	0.01	0.01	0.02	0.02	0.4	11	0.4
Wt **diabetic pregnancy/(n=19)**	0.64	3.6	0.64	0.93	2.35	1.01	34.4*‡§	434	20.8*‡
**SEM**	0.01	0.05	0.01	0.02	0.04	0.02	0.4	10	0.5
Hif1a$^{+/-}$ **non-diabetic pregnancy/(n=23)**	0.71	3.68	0.7	1.02	2.35	1.07	36.1*†§	406	22.5†
**SEM**	0.01	0.03	0.02	0.02	0.03	0.02	0.5	9	0.3
Hif1a$^{+/-}$ **diabetic pregnancy/(n=7)**	0.65	3.58	0.57	0.86	2.44	0.86	31.8*†‡	449	19.7*‡
**SEM**	0.01	0.09	0.01	0.02	0.09	0.02	1.2	14	0.5

AWTd, diastolic anterior wall thickness; LVDd, left ventricle diastolic cavity diameter; PWTd, diastolic posterior wall thickness; AWTs systolic anterior wall thickness; LVDs, left ventricle systolic cavity diameter; PWTs, systolic posterior wall thickness; FS%, fractional shortening; HR, heart rate; BW, body weight. Significant differences by post hoc Tukey's multiple-comparison test; *, $P < 0.05$ in comparison to *Wt* non-diabetic pregnancy; †, $P < 0.05$ in comparison to *Wt* diabetic pregnancy; ‡, $P < 0.05$ in comparison to *Hif1a$^{+/-}$* non-diabetic pregnancy; §, $P < 0.05$ in comparison to *Hif1a$^{+/-}$* diabetic pregnancy.
