# Supplementary material for: Adverse effects of Hif1a mutation and maternal diabetes on the offspring heart
Source: Cardiovasc Diabetol. 2018 May 12;17:68. doi: 10.1186/s12933-018-0713-0 (PMC5948854; doi:10.1186/s12933-018-0713-0)
Supplement: Supplementary file 3 — Additional file 3: Table S3. Bioinformatics classification of differentially expressed genes. [file 12933_2018_713_MOESM3_ESM.pdf]

Table S3. Bioinformatics classification of differentially expressed genes

Biological process category/Genes (N)	List of genes
**Response to stress/63**	*Ackr2, Aldh1a1, Anxa1, C1qa, C1qb, C1qc, C3ar1, C5ar1, Casp4, Ccl2, Ccl6, Ccl7, Ccl9, Cd14, Cd55, Cd9, Clasp1, Clec4n, Col3a1, Coro1a, Cotl1, Ctss, Cul4a, Cxcl1, Dab2, Dhx9, Duox1, Duoxa1, Ecm1, F13a1, Fcgr2b, Fcna, Fstl1, Icam1, Ifitm1, Il33, Impact, Itgam, Lmcd1, Loxl2, Lyz2, Malt1, Mrc1, Mylk3, Myocd, Nek1, Nlrp10, Pf4, Plek, Ptgs1, Ptprj, Rps6kb1, Serpinb6a, Setd2, Sfrp1, Srpx, Stab1, Tgfbr2, Tlr13, Txnip, Tyrobp, Vav1, Vldlr*
**Developmental process/79**	*Adra1a, Aldh1a1, Amigo2, Anxa1, C1qb, C1qc, C3ar1, C5ar1, Casp4, Ccdc141, Ccdc80, Ccl2, Ccl7, Ccl9, Cd248, Cd53, Cd9, Cep126, Clasp1, Clec3b, Col3a1, Coro1a, Ctsc, Cul4a, Cxcl1, Dab2, Dact2, Dclk1, Duox1, Duoxa1, Ecm1, Fbn1, Gas7, Hcls1, Hexa, Icam1, Ifitm1, Il33, Ildr2, Impact, Itgam, Kcnab2, Kdm3a, Kdm5a, Loxl2, Lrp1, Malt1, Mfap5, Mknk2, Mmp19, Mylk3, Myocd, Nbl1, Nek1, Nrn1, Pf4, Pi16, Pirb, Pkp2, Plek, Ptgs1, Ptprj, Rpn2, Rps6kb1, Rpsa, S100a10, Serpinb6a, Setd2, Sfrp1, Stab1, Stmn4, Syne1, Tgfbr2, Txnip, Tyrobp, Usp9x, Vav1, Vldlr, Zbtb7b*
**Metabolic process/73**	*A4galt, Adgrd1, Adra1a, Anxa1, Arel1, Asb11, Asb15, B3gat3, C1qa, C1qb, C1qc, C5ar1, Casp4, Ccl2, Ccl6, Ccl7, Ccl9, Cd55, Clec3b, Clk1, Col3a1, Ctsc, Ctss, Cul4a, Dab2, Dcaf5, Dclk1, Ddost, Dok2, Dusp27, Ecm1, Eef1g, F13a1, Fbn1, Fcna, Hcls1, Herc3, Icam1, Il33, Ildr2, Impact, Itgam, Kdm3a, Kdm5a, Loxl2, Malt1, March1, Mknk2, Mmp19, Mylk3, Myocd, Nek1, Pcolce, Pf4, Pfkfb2, Pi16, Plek, Pm20d2, Ppp1r37, Psmd1, Ptprj, Rpn2, Rps6kb1, Rpsa, Serpinb6a, Setd2, Sfrp1, Tgfbr2, Tlr13, Trim33, Txnip, Usp9x, Vldlr*
**Immune system processes/55**	*Anxa1, C1qa, C1qb, C1qc, C3ar1, C5ar1, Casp4, Ccl2, Ccl6, Ccl7, Ccl9, Cd14, Cd248, Cd300ld, Cd55, Clec4n, Col3a1, Coro1a, Ctsc, Ctss, Cul4a, Cxcl1, Dab2, Dact2, Ddost, Ecm1, Fbn1, Fcgr2b, Fcna, Fyb, Hcls1, Icam1, Ifitm1, Il33, Itgam, Kcnab2, Lmcd1, Malt1, March1, Mfap5, Mknk2, Mrc1, Myo1g, Nbl1, Nlrp10, Pf4, Pirb, Plek, Ptprj, Sfrp1, Tgfbr2, Tlr13, Tyrobp, Vav1, Zbtb7b*
**Regulation of cell proliferation/33**	*Anxa1, C3ar1, C5ar1, Ccl2, Cd248, Cd9, Coro1a, Cul4a, Cxcl1, Dab2, Dpt, Ecm1, Fcgr2b, Fgf16, Folr2, Hcls1, Ifitm1, Il33, Itgam, Loxl2, Lrp1, Malt1, Myocd, Pf4, Pkp2, Ptgs1, Ptprj, Rps6kb1, Sfrp1, Srpx, Tgfbr2, Txnip, Wdr13*
**Angiogenesis/11**	*C3ar1, C5ar1, Ccdc80, Ccl2, Ecm1, Loxl2, Mmp19, Pf4, Setd2, Stab1, Tgfbr2*
**ECM organization/10**	*Ccdc80, Ccl2, Clasp1, Col3a1, Ctss, Dpt, Loxl2, Mfap5, Mmp19, Mrc2*
**Cell death/28**	*Aldh1a1, Amigo2, Anxa1, Arel1, C5ar1, Casp4, Ccl2, Cd248, Coro1a, Ctsc, Dab2, Emp3, Fcgr2b, Hcls1, Icam1, Il33, Impact, Ivns1abp, Lrp1, Malt1, Mknk2, Myocd, Pf4, Rps6kb1, Sfrp1, Srpx, Tgfbr2, Txnip*

Table S3. Continued

Biological process category/Genes (N)	List of genes
**Cell communication/76**	*Ackr2, Adgrd1, Adra1a, Anxa1, Asb11, Asb15, C3ar1, C5ar1, Casp4, Ccl2, Ccl6, Ccl7, Ccl9, Cd14, Cd53, Cd9, Clec4n, Col3a1, Coro1a, Ctsc, Cxcl1, Dab2, Dact2, Dclk1, Dok2, Duox1, Ecm1, Fbn1, Fcgr2b, Fcna, Fgf16, Frat2, Fyb, Gpr22, Hcls1, Hexa, Icam1, Il33, Ildr2, Impact, Iqgap2, Itgam, Ivns1abp, Kdm3a, Lmcd1, Lrp1, Malt1, Mknk2, Myo1g, Myocd, Nbl1, Ncln, Olfr78, P2ry6, Pf4, Pfkfb2, Pirb, Pkp2, Plek, Ptgs1, Ptprj, Rab15, Rps6kb1, Rrad, Sfrp1, Snx13, Srpx, Stab1, Tgfbr2, Tlr13, Trim33, Txnip, Tyrobp, Usp9x, Vav1, Vldlr*
**Transport/24**	*Anxa1, Atp8a1, Atp9a, Ccl2, Cd14, Cd9, Clasp1, Coro1a, Dab2, Fcgr2b, Lrp1, Mrc1, Mrc2, Msr1, Myo1g, Osbpl6, Plek, Rab15, S100a10, Srpx, Syne1, Tgfbr2, Vav1, Vldlr*
**Others/79**	Adamtsl3, Adamtsl4, Adssl1, Aldh1a2, Ankrd28, Arpc1b, Arpc3, Atf6b, Axl, Blk, Cadm3, Capg, Ccser2, Cd320, Cdk2ap2, Cfp, Chd4, Clpx, Cmah, Cpn2, Creld1, Ctsa, Ctsz, Dhdh, Efhd2, Emc10, Emilin2, Fbln1, Fbln2, Fgl2, Fmr1nb, Fxyd5, Fyco1, G0s2, Gusb, Hck, Hn1, Igfbp4, Itgb2, Kap, Laptm5, Lbp, Luzp2, Lyve1, Mat2a, Mmp9, Mpeg1, Mpped2, Ms4a6d, Myh11, Ncf1, Ncf4, Nxpe5, Pacsin2, Pcf11, Pknox2, Pla1a, Plekhh1, Plod3, Ptchd3, Ptgis, Rpl8, Sacs, Scin, Sla, Slc35e2, Slc7a7, Tcn2, Timp1, Tmem245, Ttc39b, Ubn2, Vwf, Wfdc17, Xpo4, Zbtb11, Zfp397, Zfp442, Zfp655
