# Supplementary material for: Adverse effects of Hif1a mutation and maternal diabetes on the offspring heart
Source: Cardiovasc Diabetol. 2018 May 12;17:68. doi: 10.1186/s12933-018-0713-0 (PMC5948854; doi:10.1186/s12933-018-0713-0)
Supplement: Supplementary file 4 — Additional file 4: Table S4. List of genes in Venn diagram in Fig. 3. [file 12933_2018_713_MOESM4_ESM.pdf]

Table S4: List of genes in Venn diagram in Fig. 3.

Group/Genes (N)	List of genes
***Hif1a*$^{+/-}$ diabetic pregnancy/(135)**	*Itgb2, Cfp, Timp1, Vwf, Scin, Axl, Pla1a, Hck, Cadm3, Fbln1, G0s2, Fxyd5, Aldh1a2, Blk, Adamtsl4, Ncf1, Lbp, Cmah, Igfbp4, Mmp9, Ptgis, Myh11, Ccl6, Ccl9, Plek, Myo1g, Myocd, Rab15, Iqgap2, Xpo4, Stmn4, Dok2, Fyb, Dab2, Sla, Zbtb11, Ccdc80, Fstl1, Hcls1, Cpn2, Clec4n, Pi16, Emilin2, Zfp397, Ms4a6d, Il33, Msr1, Ptprj, Ifitm1, Clec3b, Gsta3, Col3a1, Cd55, Dpt, Fcgr2b, Mrc1, Fcna, Fbn1, Tspan18, Duoxa1, Dclk1, Ecm1, Laptm5, Kcnab2, Pf4, Cxcl1, Arpc1b, Mfap5, Ctsc, Tyrobp, Coro1a, Itgam, Lyve1, Cotl1, Rrad, Tgfbr2, Malt1, Folr2, Kap, Gas7, Duox1, Casp4, Tlr13, Vav1, Loxl2, Cd300ld, Ccl7, Ccl2, Pknox2, March1, C1qa, C1qc, C1qb, Ctss, F13a1, Nrn1, Ptchd3, Fgl2, Emp3, Lrp1, C3ar1, Ildr2, Cd53, Nbl1, Pcf11, Slc35e2, Stab1, Olfr78, Adgrd1, Gpr22, Ackr2, Adra1a, Mpeg1, Ptgs1, Nxpe5, Frat2, A4galt, Amigo2, P2ry6, Dact2, C5ar1, Cdh20, Cd14, Kdm3a, Pm20d2, Cd248, Capg, Pirb, Fmr1nb, Luzp2, Lyz2, Wfdc17, Adamtsl3, Ncf4, Srpx*
***Wt* diabetic pregnancy/(39)**	*Scin, Axl, Fxyd5, Aldh1a2, Ncf1, Ptgis, Ccl6, Ccl9, Mknk2, Rab15, Stmn4, Fyb, Sla, Ms4a6d, Il33, Msr1, Gsta3, Fbn1, Tspan18, Cxcl1, Lyve1, Tsc22d3, Kap, Tlr13, Ccl2, March1, Txnip, Ctss, Fgl2, Cd53, Olfr78, Frat2, Amigo2, Cdh20, Aldh1a1, Fmr1nb, Cytl1, Luzp2, Trp53i11*
***Hif1a*$^{+/-}$ non-diabetic pregnancy/(17)**	*Mmp9, Rab15, Hif1a, Duoxa1, Itgam, Kap, Ccl7, Ccl2, Nrn1, Ptchd3, Olfr78, Ackr2, Frat2, Cdh20, Plekhh1, Fmr1nb, Zfp442*
***Hif1a*$^{+/-}$ diabetic pregnancy + *Wt* diabetic pregnancy/(26)**	*Ctss, Lyve1, Il33, Ccl6, Stmn4, Tspan18, Gsta3, Fgl2, Scin, Ccl9, Aldh1a2, Axl, Ms4a6d, Amigo2, Sla, Ptgis, Msr1, Cd53, Tlr13, Fbn1, Fyb, Luzp2, Cxcl1, Ncf1, March1, Fxyd5*
***Hif1a*$^{+/-}$ diabetic pregnancy + *Hif1a*$^{+/-}$ non-diabetic pregnancy/(7)**	*Ackr2, Duoxa1, Nrn1, Mmp9, Ccl7, Itgam, Ptchd3*
***Hif1a*$^{+/-}$ diabetic prgnancy + *Wt* diabetic pregnancy + *Hif1a*$^{+/-}$ non-diabetic pregnancy/(7)**	*Frat2, Cdh20, Olfr78, Ccl2, Kap, Fmr1nb, Rab15*

30%-fold change threshold to *Wt* non-diabetic pregnancy was applied, genes with great variability were excluded from analysis.
