# Supplementary material for: Adverse effects of Hif1a mutation and maternal diabetes on the offspring heart
Source: Cardiovasc Diabetol. 2018 May 12;17:68. doi: 10.1186/s12933-018-0713-0 (PMC5948854; doi:10.1186/s12933-018-0713-0)
Supplement: Supplementary file 5 — Additional file 5: Table S5. List of differentially expressed genes with fold change ≥ 30%. [file 12933_2018_713_MOESM5_ESM.pdf]

Table S5: List of differentially expressed genes with fold change ≥ 30%

Group/Genes (N)	List of genes
Upregulated genes (111)	*Itgb2, Cfp, Timp1, Vwf, Axl, Pla1a, Hck, Cadm3, Fbln1, Fxyd5, Aldh1a2, Adamtsl4, Ncf1, Lbp, Cmah, Igfbp4, Ptgis, Ccl6, Ccl9, Plek, Myo1g, Rab15, Stmn4, Dok2, Fyb, Dab2, Sla, Ccdc80, Fstl1, Hcls1, Clec4n, Pi16, Emilin2, Ms4a6d, Il33, Msr1, Ptprj, Ifitm1, Clec3b, Gsta3, Col3a1, Cd55, Dpt, Fcgr2b, Mrc1, Fcna, Duoxa1, Dclk1, Ecm1, Laptm5, Kcnab2, Pf4, Cxcl1, Arpc1b, Mfap5, Ctsc, Tyrobp, Coro1a, Lyve1, Cotl1, Rrad, Tgfbr2, Folr2, Gas7, Duox1, Casp4, Tlr13, Vav1, Loxl2, Cd300ld, Pknox2, March1, C1qa, C1qc, C1qb, Ctss, F13a1, Fgl2, Emp3, Lrp1, C3ar1, Ildr2, Cd53, Nbl1, Stab1, Adgrd1, Mpeg1, Ptgs1, Nxpe5, Frat2, A4galt, Amigo2, P2ry6, Dact2, C5ar1, Cd14, Cd248, Capg, Pirb, Lyz2, Wfdc17, Adamtsl3, Ncf4, Srpx, Mknk2, Tsc22d3, Aldh1a1, Cytl1, Hif1a, Plekhh1, Zfp442*
Downregulated genes (25)	*Scin, G0s2, Blk, Myh11, Myocd, Iqgap2, Xpo4, Zbtb11, Cpn2, Zfp397, Tspan18, Malt1, Nrn1, Ptchd3, Pcf11, Slc35e2, Olfr78, Gpr22, Adra1a, Cdh20, Kdm3a, Pm20d2, Luzp2, Txnip, Trp53i11*
Differentially regulated genes (8)	*Mmp9, Itgam, Kap, Ccl7, Ccl2, Ackr2, Fmr1nb, Fbn1*
