# Supplementary material for: Adverse effects of Hif1a mutation and maternal diabetes on the offspring heart
Source: Cardiovasc Diabetol. 2018 May 12;17:68. doi: 10.1186/s12933-018-0713-0 (PMC5948854; doi:10.1186/s12933-018-0713-0)
Supplement: Supplementary file 6 — Additional file 6: Table S6. The list of HIF-1 signaling target genes. [file 12933_2018_713_MOESM6_ESM.pdf]

Table S6: The list of HIF-1 signaling target genes

**Category**	N genes/ % of 135	
Direct HIF-1 target genes	9/6.7%	*Adgrd1, Axl, Cd55, Cfp, Kdm3a, Loxl2, Lrp1, Pcf11, Sla*
Gene expression linked to *Hif1a*	18/13.3%	*Aldh1a2, Capg, Cd248, Col3a1, Coro1a, Cxcl1, Dclk1, Ifitm1, Il33, Lyve1, Msr1, Myh11, Ptgis, Ptgs1, Ptprj, Srpx, Tgfbr2, Timp1*
Gene expression linked to hypoxia	42/31.1%	*Adra1a, Amigo2, C1qa, C1qb, Ccl2, Ccl7, Ccl9, Cd53, Cpn2, Ctsc, Ctss, Dpt, Ecm1, Emp3, F13a1, Fbln1, Fbn1, Fcgr2b, Fgl2, Folr2, Frat2, Fstl1, Fxyd5, Fyb, G0s2, Gas7, Gsta3, Igfbp4, Itgb2, Kap, Lbp, Mmp9, Mpeg1, Myocd, Ncf1, Nrn1, Pirb, Rab15, Rrad, Stab1, Vav1, Vwf*
Predicted HIF-1 target genes	12/8.9%	*C5ar1, Capg, Ccdc80, Ctss, Fmr1nb, Fyb, Itgb2, Mmp9, Myocd, Ptgis, Rab15, Vwf*
Genes from the families with predicted HIF-1 targets	62/45.9%	*Ackr2, Adamtsl3, Adamtsl4, Adgrd1, Aldh1a2, Amigo2, Arpc1b, C1qa, C1qb, C1qc, Casp4, Ccl2, Ccl6, Ccl7, Ccl9, Cd14, Cd248, Cd300ld, Cd53, Cd55, Cdh20, Clec3b, Clec4n, Col3a1, Coro1a, Ctsc, Cxcl1, Ecm1, Emilin2, Emp3, Fbln1, Fcna, Frat2, Fxyd5, Gas7, Gpr22, Gsta3, Ifitm1, Il33, Itgam, Kcnab2, Kdm3a, Lbp, Mfap5, Mrc1, Ms4a6d, Myh11, Myo1g, Nxpe5, Olfr78, P2ry6, Plek, Ptchd3, Ptgs1, Ptprj, Slc35e2, Tgfbr2, Tlr13, Vav1, Wfdc17, Zbtb11, Zfp397*

The list of genes with fold change ≤ 0.3 in *Hif1a+/-* offspring exposed to maternal diabetes (N=135) was used for manual literature search for direct targets of HIF-1 signalling, genes affected by alteration in the *Hif1a* expression, and hypoxia. Harmonizome database was used for the search of predicted targets and genes from families of genes with predicted targets based on binding site motifs.
