# Supplementary material for: Adverse effects of Hif1a mutation and maternal diabetes on the offspring heart
Source: Cardiovasc Diabetol. 2018 May 12;17:68. doi: 10.1186/s12933-018-0713-0 (PMC5948854; doi:10.1186/s12933-018-0713-0)
Supplement: Supplementary file 7 — Additional file 7: Table S7. The list of the references for genes linked to HIF-1 signalling by manual literature search. [file 12933_2018_713_MOESM7_ESM.pdf]

Table S7: The list of the references for genes linked to HIF-1 signalling by manual literature search

Symbol	Name	PubMed ID
Adgrd1	adhesion G protein-coupled receptor D1	PMID: 27775701
Adra1a	adrenergic receptor, alpha 1a	PMC2951593
Aldh1a2	aldehyde dehydrogenase family 1, subfamily A2	PMID: 26220168
Amigo2	adhesion molecule with Ig like domain 2	PMID: 20848564
Axl	AXL receptor tyrosine kinase	PMID: 25187556
Blk	B lymphoid kinase	PMID: 26626568
C1qa	complement component 1, q subcomponent, alpha polypeptide	PMID: 28858246
C1qb	complement component 1, q subcomponent, beta polypeptide	PMID: 28858246
Capg	capping protein (actin filament), gelsolin-like	PMID: 19188659
Ccl2	chemokine (C-C motif) ligand 2	PMID: 14734750
Ccl7	chemokine (C-C motif) ligand 7	PMID: 20729179
Ccl9	chemokine (C-C motif) ligand 9	PMID: 15625408
Cd248	CD248 antigen, endosialin	PMID: 18813310
Cd53	CD53 antigen	PMID: 15625408
Cd55	CD55 molecule, decay accelerating factor for complement	PMID: 15923405
Col3a1	collagen, type III, alpha 1	PMID: 19790048
Coro1a	coronin, actin binding protein 1A	PMID: 16565084
Cpn2	carboxypeptidase N, polypeptide 2	PMID: 24867948
Ctsc	cathepsin C	PMID: 19454749
Ctss	cathepsin S	PMID: 23816886
Cxcl1	chemokine (C-X-C motif) ligand 1	PMID: 19454749
Dclk1	doublecortin-like kinase 1	PMID: 27320910
Dpt	dermatopontin	PMID: 23262218
Ecm1	extracellular matrix protein 1	PMID: 22495609
Emp3	epithelial membrane protein 3	PMID: 15100389
F13a1	coagulation factor XIII, A1 subunit	PMID: 27211559
Fbln1	fibulin 1	PMID: 22495609
Fbn1	fibrillin 1	PMID: 29215599
Fcgr2b	Fc receptor, IgG, low affinity IIb	PMID: 16849508
Fgl2	fibrinogen-like protein 2	PMID: 20416888
Folr2	folate receptor 2 (fetal)	PMID: 26209622
Frat2	frequently rearranged in advanced T cell lymphomas 2	PMID: 19725949
Fstl1	follistatin-like 1	PMID: 28361925
Fxyd5	FXYD domain-containing ion transport regulator 5	PMC2743718
Fyb	FYN binding protein	PMID: 24114211
G0s2	G0/G1 switch gene 2	PMID: 24344269
Gas7	growth arrest specific 7	PMID: 16849508
Gsta3	glutathione S-transferase, alpha 3	PMID: 21447374
Ifitm1	interferon induced transmembrane protein 1	PMID: 20003295
Igfbp4	insulin-like growth factor binding protein 4	PMID: 18366759

Table S7: Continued

Symbol	Name	PubMed ID
Il33	interleukin 33	PMID: 23967327
Itgb2	integrin beta 2	PMID: 19740309
Kap	kidney androgen regulated protein	PMID: 15263007
Kdm3a	lysine (K)-specific demethylase 3A	PMID: 22645302
Lbp	lipopolysaccharide binding protein	PMID: 18984585
Loxl2	lysyl oxidase-like 2	PMID: 22645302
Lrp1	low density lipoprotein receptor-related protein 1	PMID: 21454812
Lyve1	lymphatic vessel endothelial hyaluronan receptor 1	PMID: 26828208
Mmp9	matrix metallopeptidase 9	PMID: 18097553
Mpeg1	macrophage expressed gene 1	PMID: 18469115
Msr1	macrophage scavenger receptor 1	PMID: 19641936
Myh11	myosin, heavy polypeptide 11, smooth muscle	PMID: 27456656
Myocd	myocardin	PMID: 27144530, PMID: 19098903
Ncf1	neutrophil cytosolic factor 1	PMID: 18314479
Nrn1	neuritin 1	PMID: 19491311
Pcf11	PCF11 cleavage and polyadenylation factor subunit	PMID: 19386601, PMID: 17532074
Pirb	paired Ig-like receptor B	PMID: 22580685
Ptgis	prostaglandin I2 (prostacyclin) synthase	PMID: 15374877
Ptgs1	prostaglandin-endoperoxide synthase 1	PMID: 15374877
Ptprj	protein tyrosine phosphatase, receptor type, J	PMID: 23007793
Rab15	RAB15, member RAS oncogene family	PMID: 17828398
Rrad	Ras-related associated with diabetes	PMID: 25114038
Sla	src-like adaptor	PMID: 21447827
Srpx	sushi-repeat-containing protein	PMID: 16565084
Stab1	stabilin 1	PMID: 16849508
Tgfbr2	transforming growth factor, beta receptor II	PMID: 26146544
Timp1	tissue inhibitor of metalloproteinase 1	PMID: 16711028
Vav1	vav 1 oncogene	PMID: 16849508
Vwf	Von Willebrand factor	PMID: 23580145
