# Supplementary material for: Adverse effects of Hif1a mutation and maternal diabetes on the offspring heart
Source: Cardiovasc Diabetol. 2018 May 12;17:68. doi: 10.1186/s12933-018-0713-0 (PMC5948854; doi:10.1186/s12933-018-0713-0)
Supplement: Supplementary file 8 — Additional file 8: Table S8. Bioinformatics enrichment analysis using gProfiler: g:GOST Gene Group Functional Profiling tool. [file 12933_2018_713_MOESM8_ESM.pdf]

Table S8. Bioinformatics enrichment analysis using gProfiler: g:GOST Gene Group Functional Profiling tool.

	Enriched biological process category/Hits (N)	List of genes
Wt diabetic pregnancy	Inflammatory response/(10) P=0.000151	*Axl, Ncf1, Ptgis, Ccl6, Ccl9, Il33, Cxcl1, Tlr13, Ccl2, Ctss*
	Immune system process/(16) P=0.00173	*Scin, Axl, Ncf1, Ccl6, Ccl9, Mknk2, Fyb, Sla, Il33, Fbn1, Cxcl1, Tsc22d3, Tlr13, Ccl2, March1, Ctss*
Hif1a$^{+/-}$ diabetic pregnancy	Immune system process/(54) P=2.72E-16	*Itgb2, Cfp, Scin, Axl, Hck, Blk, Ncf1, Lbp, Mmp9, Ccl6, Ccl9, Plek, Myo1g, Fyb, Sla, Hcls1, Clec4n, Il33, Ptprj, Ifitm1, Col3a1, Cd55, Mrc1, Fcna, Fbn1, Ecm1, Kcnab2, Pf4, Cxcl1, Mfap5, Ctsc, Tyrobp, Coro1a, Tgfbr2, Malt1, Casp4, Tlr13, Vav1, Cd300ld, Ccl7, Ccl2, March1, C1qa, C1qc, C1qb, Ctss, Lrp1, C3ar1, Nbl1, Dact2, C5ar1, Cd14, Cd248, Pirb*
	Developmental process/(61) P=0.00226	*Itgb2, Timp1, Scin, Axl, Hck, Fbln1, Aldh1a2, Blk, Adamtsl4, Mmp9, Ptgis, Myh11, Ccl9, Plek, Myocd, Stmn4, Dab2, Sla, Hcls1, Il33, Msr1, Ifitm1, Clec3b, Gsta3, Col3a1, Fbn1, Duoxa1, Dclk1, Ecm1, Kcnab2, Pf4, Cxcl1, Mfap5, Ctsc, Tyrobp, Coro1a, Tgfbr2, Malt1, Duox1, Casp4, Vav1, Loxl2, Ccl7, Ccl2, C1qc, C1qb, Nrn1, Lrp1, C3ar1, Ildr2, Cd53, Nbl1, Stab1, Adra1a, Ptgs1, Amigo2, Dact2, C5ar1, Kdm3a, Cd248, Pirb*
	Response to stimulus/(90) P=3.38E-6	*Itgb2, Cfp, Timp1, Vwf, Axl, Hck, Fbln1, G0s2, Aldh1a2, Blk, Ncf1, Lbp, Igfbp4, Mmp9, Ptgis, Ccl6, Ccl9, Plek, Myo1g, Myocd, Iqgap2, Dok2, Fyb, Dab2, Sla, Fstl1, Hcls1, Clec4n, Il33, Msr1, Ptprj, Ifitm1, Clec3b, Gsta3, Col3a1, Cd55, Mrc1, Fcna, Fbn1, Tspan18, Duoxa1, Dclk1, Ecm1, Laptm5, Pf4, Cxcl1, Arpc1b, Ctsc, Tyrobp, Coro1a, Itgam, Lyve1, Cotl1, Rrad, Tgfbr2, Malt1, Duox1, Casp4, Tlr13, Vav1, Loxl2, Ccl7, Ccl2, March1, C1qa, C1qc, C1qb, Ctss, F13a1, Lrp1, C3ar1, Ildr2, Cd53, Nbl1, Stab1, Olfr78, Adgrd1, Gpr22, Ackr2, Adra1a, Ptgs1, Frat2, P2ry6, Dact2, C5ar1, Cd14, Kdm3a, Pirb, Lyz2, Srpx*
	Cell communication/(69) P=0.00108	*Itgb2, Timp1, Axl, Hck, Fbln1, G0s2, Aldh1a2, Blk, Ncf1, Lbp, Igfbp4, Mmp9, Ptgis, Ccl6, Ccl9, Plek, Myo1g, Myocd, Iqgap2, Dok2, Fyb, Dab2, Sla, Hcls1, Clec4n, Il33, Ptprj, Col3a1, Mrc1, Fcna, Fbn1, Tspan18, Dclk1, Ecm1, Pf4, Cxcl1, Ctsc, Tyrobp, Coro1a, Itgam, Lyve1, Rrad, Tgfbr2, Malt1, Duox1, Casp4, Tlr13, Vav1, Ccl7, Ccl2, Lrp1, C3ar1, Ildr2, Cd53, Nbl1, Stab1, Olfr78, Adgrd1, Gpr22, Ackr2, Adra1a, Frat2, P2ry6, Dact2, C5ar1, Cd14, Kdm3a, Pirb, Srpx*

Table S8. Continued

	Enriched biological process category/Hits (N)	List of genes
Hif1a$^{+/-}$ diabetic pregnancy	Apoptotic process/(26) P=0.0455	*Timp1, Scin, Axl, Hck, G0s2, Aldh1a2, Adamtsl4, Ncf1, Mmp9, Ptgis, Myocd, Dab2, Hcls1, Il33, Pf4, Ctsc, Coro1a, Tgfbr2, Malt1, Casp4, Ccl2, Lrp1, Amigo2, C5ar1, Cd248, Srpx*
	Regulation of angiogenesis/(9) P=0.0244	*Itgb2, Ptgis, Ecm1, Pf4, Tgfbr2, Ccl2, C3ar1, Stab1, C5ar1*
	Cell proliferation/(30) P=0.00159	*Timp1, Scin, Hck, Fbln1, Aldh1a2, Blk, Ncf1, Igfbp4, Mmp9, Myocd, Sla, Hcls1, Il33, Ptprj, Ifitm1, Dpt, Ecm1, Pf4, Cxcl1, Coro1a, Tgfbr2, Malt1, Folr2, Loxl2, Ccl2, Lrp1, Ptgs1, C5ar1, Cd248, Srpx*
	Endocytosis/(18) P=0.00025	*Axl, Hck, Lbp, Myo1g, Dab2, Msr1, Mrc1, Coro1a, Tgfbr2, Vav1, Loxl2, Ccl2, Lrp1, Stab1, Ackr2, Cd14, Ncf4, Srpx*
	Response to stress/(55) P=1.84E-09	*Itgb2, Cfp, Timp1, Vwf, Axl, Hck, Fbln1, Blk, Ncf1, Lbp, Igfbp4, Mmp9, Ptgis, Ccl6, Ccl9, Plek, Myocd, Sla, Fstl1, Clec4n, Il33, Ifitm1, Col3a1, Cd55, Mrc1, Fcna, Duoxa1, Ecm1, Pf4, Cxcl1, Tyrobp, Coro1a, Cotl1, Tgfbr2, Malt1, Duox1, Casp4, Tlr13, Vav1, Loxl2, Ccl7, Ccl2, C1qa, C1qc, C1qb, Ctss, F13a1, C3ar1, Stab1, Ackr2, Ptgs1, C5ar1, Cd14, Lyz2, Srpx*
	Leukocyte migration/(17) P=9.51E-09	*Itgb2, Lbp, Mmp9, Ccl6, Ccl9, Myo1g, Il33, Ecm1, Pf4, Cxcl1, Coro1a, Vav1, Ccl7, Ccl2, C3ar1, Nbl1, C5ar1*
	Extracellular matrix organization/(9) P=0.0121	*Fbln1, Adamtsl4, Mmp9, Myh11, Ccdc80, Col3a1, Dpt, Loxl2, Ctss*
