# Supplementary material for: Adverse effects of Hif1a mutation and maternal diabetes on the offspring heart
Source: Cardiovasc Diabetol. 2018 May 12;17:68. doi: 10.1186/s12933-018-0713-0 (PMC5948854; doi:10.1186/s12933-018-0713-0)
Supplement: Supplementary file 9 — Additional file 9: Table S9. Bioinformatics mammalian phenotype ontology enrichment analysis using MouseMine Analysis Tools. [file 12933_2018_713_MOESM9_ESM.pdf]

Table S9. Bioinformatics mammalian phenotype ontology enrichment analysis using MouseMine Analysis Tools.

Mammalian Phenotype Ontology Enrichment Category	Holm-Bonferroni	Number of genes
abnormal innate immunity [MP:0002419]	3.93E-10	25
abnormal blood vessel physiology [MP:0000249]	5.85E-06	17
abnormal vascular smooth muscle physiology [MP:0005595]	0.002573	9
abnormal immune system physiology [MP:0001790]	2.10E-08	51
immune system phenotype [MP:0005387]	2.74E-07	60
homeostasis/metabolism phenotype [MP:0005376]	0.000693	63
