# Supplementary material for: Adverse effects of Hif1a mutation and maternal diabetes on the offspring heart
Source: Cardiovasc Diabetol. 2018 May 12;17:68. doi: 10.1186/s12933-018-0713-0 (PMC5948854; doi:10.1186/s12933-018-0713-0)
Supplement: Supplementary file 10 — Additional file 10: Figure S1. RNAseq data validation by RT-qPCR. The values are mean ± SEM (n = 3/group for RNA-Seq, n = 8/group for RT-qPCR). [file 12933_2018_713_MOESM10_ESM.pdf]

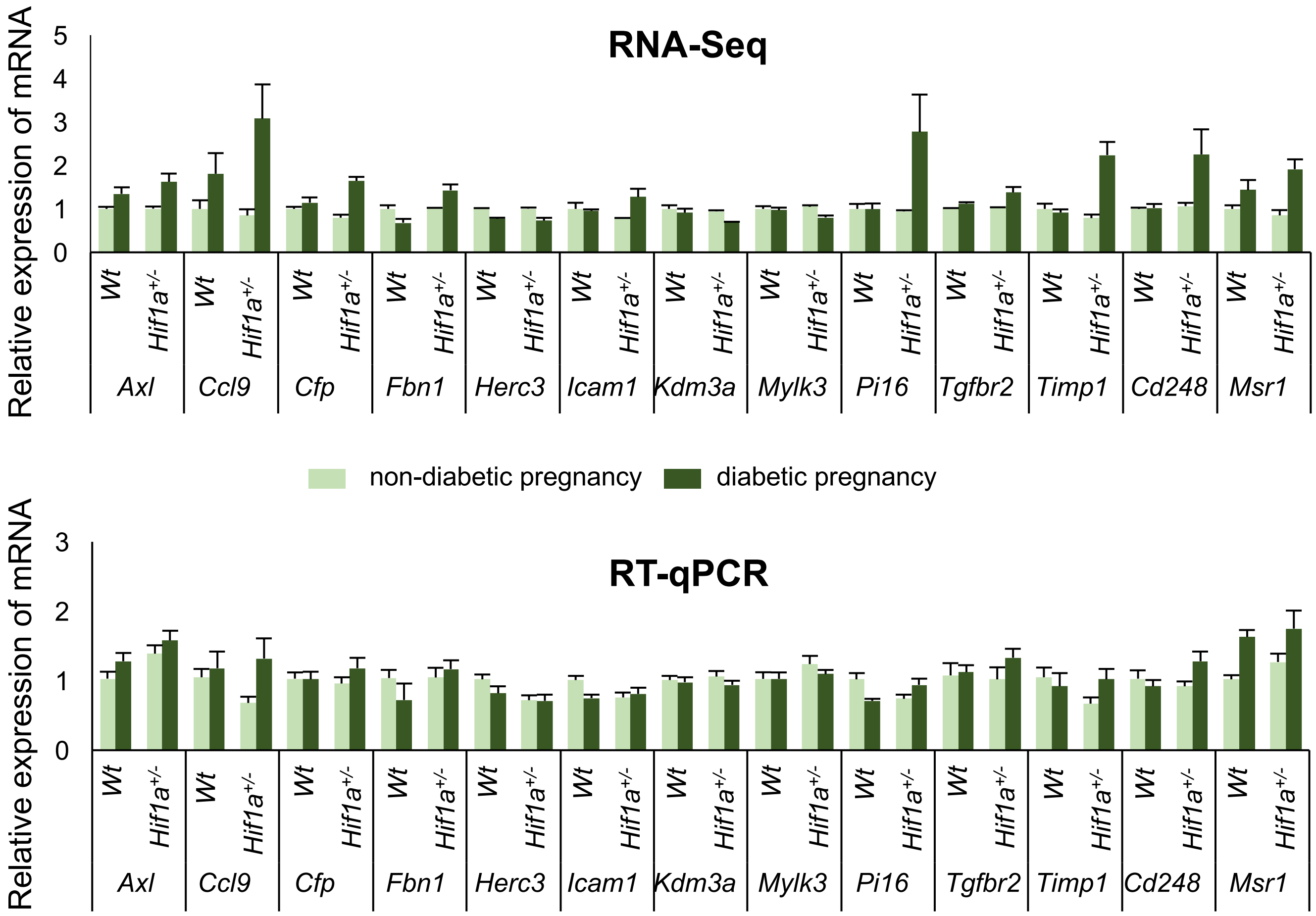

Figure S1. RNAseq data validation by RT-qPCR. The values are mean ± SEM (n = 3/group for RNA-Seq, n = 8/group for RT-qPCR).
