# Supplementary material for: Adverse effects of Hif1a mutation and maternal diabetes on the offspring heart
Source: Cardiovasc Diabetol. 2018 May 12;17:68. doi: 10.1186/s12933-018-0713-0 (PMC5948854; doi:10.1186/s12933-018-0713-0)
Supplement: Supplementary file 11 — Additional file 11: Figure S2. Quantification of TUNEL+ apoptotic cells per mm2 of the RV myocardium (a) and septum (b). The values are mean ± SEM (n = 4 individuals/3 sections/group). Two-way ANOVA indicating a significant effect of diabetes (RV: P < 0.0001; septum: P < 0.0001) followed by post hoc Tukey’s multiple-comparison test, **P < 0.01, ***P < 0.001, **** P < 0.0001. [file 12933_2018_713_MOESM11_ESM.pdf]

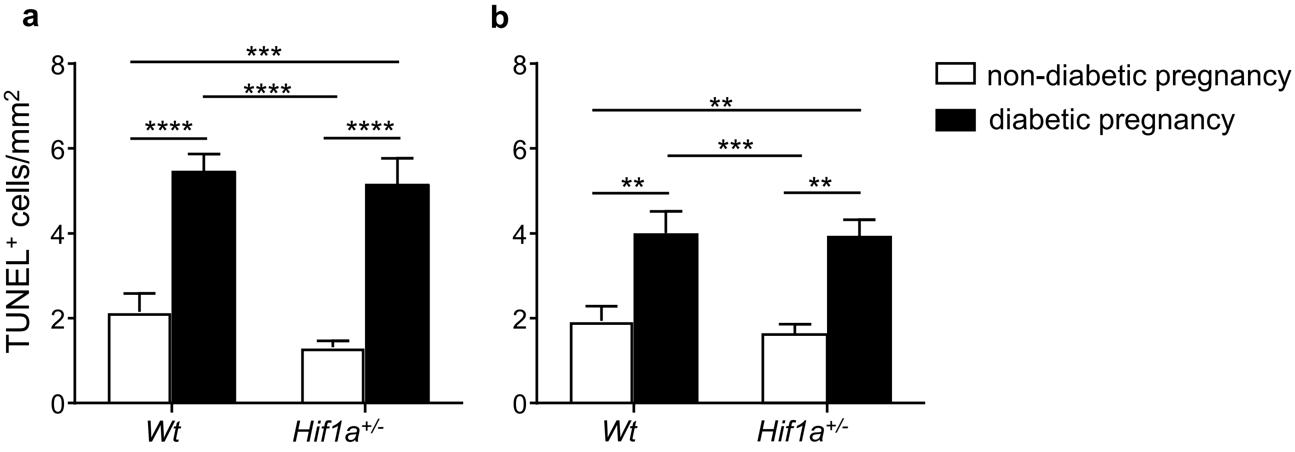

Figure S2. Quantification of TUNEL$^+$ apoptotic cells per mm^2 of the RV myocardium (**a**) and septum (**b**). The values are mean ± SEM (n = 4 individuals/3 sections/group). Two-way ANOVA indicating a significant effect of diabetes (RV: $P < 0.0001$; septum: $P < 0.0001$) followed by post hoc Tukey's multiple-comparison test, **$P < 0.01$, ***$P < 0.001$, **** $P < 0.0001$.
