# Supplementary material for: Adverse effects of Hif1a mutation and maternal diabetes on the offspring heart
Source: Cardiovasc Diabetol. 2018 May 12;17:68. doi: 10.1186/s12933-018-0713-0 (PMC5948854; doi:10.1186/s12933-018-0713-0)
Supplement: Supplementary file 12 — Additional file 12: Figure S3. PECAM-1 expression in the LV. Representative images of staining of PECAM-1 (red) with Hoechst stained cell nuclei (blue) in the LV of 12 week-old offspring (a-d). Scale bar = 50 μm. e-h: Delineated PECAM+ area in the myocardium using Adobe Photoshop. Quantification of PECAM-1 staining determined as a percentage of positive area in the field of view by ImageJ (i). The values are mean ± SEM (n = 4). Statistical significance assessed by two-way ANOVA: genotype effect P = 0.0302, followed by post hoc Tukey’s multiple-comparison test with no significant result. [file 12933_2018_713_MOESM12_ESM.pdf]

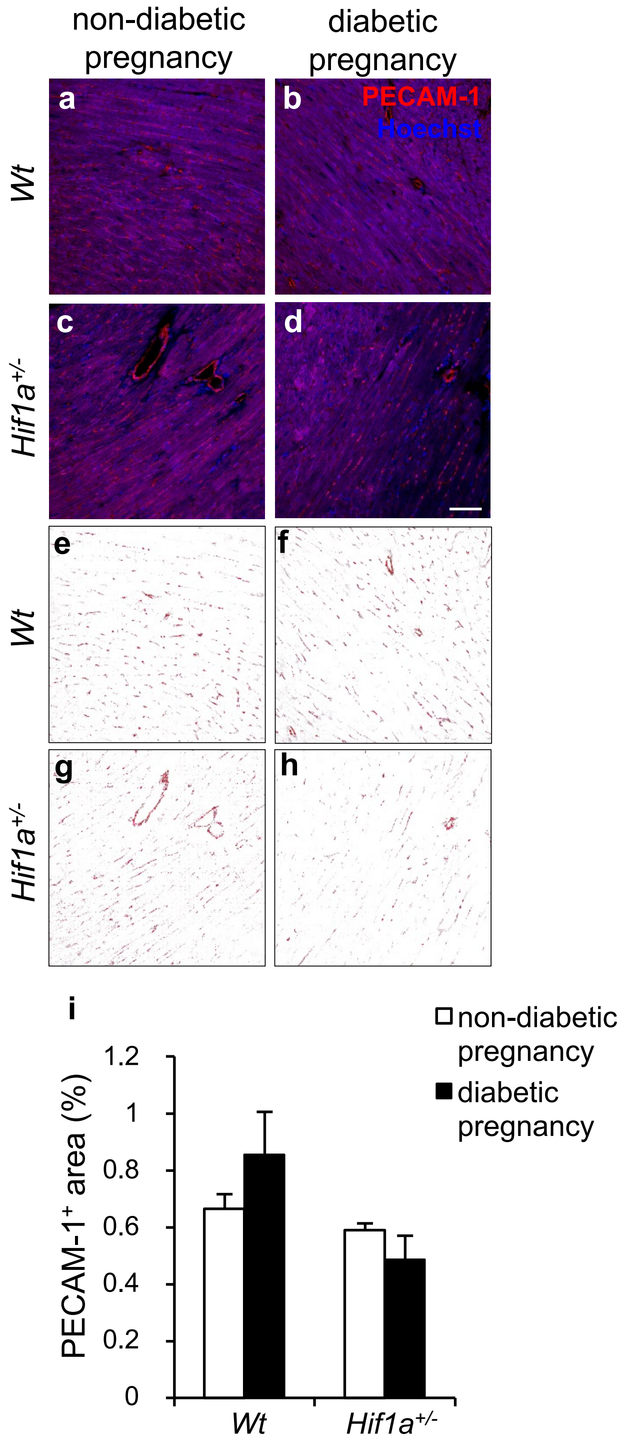

Figure S3. PECAM-1 expression in the LV. Representative images of staining of PECAM-1 (red) with Hoechst stained cell nuclei (blue) in the LV of 12 week-old offspring (**a-d**). Scale bar = 50 µm. **e-h**: Delineated PECAM^{+} area in the myocardium using Adobe Photoshop. Quantification of PECAM-1 staining determined as a percentage of positive area in the field of view by ImageJ (**i**). The values are mean ± SEM (n = 4). Statistical significance assessed by two-way ANOVA: genotype effect P = 0.0302, followed by post hoc Tukey's multiple-comparison test with no significant result.
